# Model-Based Meta-Analysis on the Efficacy of Biologics and Small Targeted Molecules for Crohn’s Disease

**DOI:** 10.3389/fimmu.2022.828219

**Published:** 2022-03-17

**Authors:** Boran Yu, Libo Zhao, Siyao Jin, Huan He, Jing Zhang, Xiaoling Wang

**Affiliations:** ^1^ Clinical Research Center, Beijing Children’s Hospital, Capital Medical University, National Center for Children’s Health, Beijing, China; ^2^ School of Pharmacy, Capital Medical University, Beijing, China; ^3^ Department of Gastroenterology, Beijing Children’s Hospital, Capital Medical University, National Center for Children’s Health, Beijing, China

**Keywords:** model-based meta-analysis, Crohn’s disease, biologics, small targeted molecules, relative efficacy

## Abstract

Information on comparative drug efficacy is of great importance for drug development as well as clinical practice. Up to now, the relative efficacy of biologics and small targeted molecules for Crohn’s disease (CD) remains unclear. The objective of this study was to quantify the relative efficacy of investigational and approved biological treatments for CD measured in Crohn’s Disease Activity Index (CDAI), Inflammatory Bowel Disease Questionnaire (IBDQ), and C-reactive protein (CRP). The analysis dataset was composed of summary-level data from 46 trials, containing 12,846 patients, with treatment of 24 drugs. Six mathematical models with non-parametric placebo estimations were developed to describe the time course and dose–response of six efficacy measures. The effects of covariate were further evaluated. Time–response relationships were found in outcomes measured in CDAI. The patients’ age, disease duration, baseline CDAI, and CRP showed an impact on the efficacy. Model simulations were performed to compare the efficacies across different drugs. The most achievement in clinical remission (defined as CDAI less than 150) and clinical response (defined as the reduction in CDAI for 100 or 70) was observed in the simulation for PF-04236921 and infliximab, respectively. The most improvement in IBDQ was shown in tofacitinib. In general, tumor necrosis factor (TNF)-α inhibitors were the most effective biologics, and the highest efficacy of small targeted molecules was observed in janus kinase (JAK) inhibitors. These findings have important implications for clinical practice in CD.

## Introduction

Crohn’s disease (CD) is a chronic inflammatory disease of the gastrointestinal tract, with symptoms like chronic abdominal pain, diarrhea, obstruction, and perianal lesions (1–4). Worldwide, the estimated incidence of CD ranges from 0.58 to 20.2 cases per 100,000 person-years, while the prevalence amount to 50–322 per 100,000 persons (3, 5). Medical therapy used to treat CD includes the categories of 5-aminosalicylates (5-ASA), antibiotics, corticosteroids, immunomodulators, and biologics (6). Biologics are by far the most potent treatment for CD (3) and are strongly recommended for patients with moderate-to-severe CD who failed to respond to conventional therapy (6, 7). Six biologic agents have been approved for the treatment of CD, and a number of biologics and small targeted molecules are under investigation. However, no specific drug is preferred in the guidelines (6–8), and physicians often choose therapies on the basis of personal experience due to the deficiency of head-to-head comparison (3).

Several meta-analyses and network meta-analyses have been conducted for the potential difference between treatments for CD (2, 9–11). However, these researches focused on the absolute efficacy, without considering the placebo effect, and the relative clinical efficacy remains unknown. Besides, in most studies, the assessments of drug efficacy were only based on the end-of-study results without considering the time course. Furthermore, the efficacies of different doses were pooled as summary-level data, which led to the inadequate utilization of available data. The influence of baseline characteristics on efficacy has been researched in only several drugs by previous studies (12–14), and the influence on most drugs still has not been measured.

Model-based meta-analysis (MBMA) is an extension of traditional meta-analysis (15), representing a framework for assessing the magnitude of the treatment response and its time course (16). The introduction of dose–response and time-course models, as well as the influence of baseline characteristics, makes it possible to incorporate all studies and treatments into the analysis and to utilize the totality of the information learned from trials (17). In addition, predictions can be made for all regimens of interest in an identical study design for a more valid comparison between treatments (15, 17). Therefore, it could offer a more informative view of the data in contrast to the traditional meta-analysis (15).

The main objective of this study is to use an MBMA approach to accurately quantify the relative efficacy and onset across different biologics and small targeted molecules, including those approved and undergoing investigation. The efficacy is measured by six outcomes reported in the clinical trials of CD: absolute Crohn’s Disease Activity Index (CDAI) score of less than 150 (CDAI150), reduction of at least 70 points in the CDAI score (CDAI-70), reduction of at least 100 points in the CDAI score (CDAI-100), change from baseline in CDAI (18), C-reactive protein (CRP) (19), and Inflammatory Bowel Disease Questionnaire (IBDQ) (20).

## Methods

### Data Development

The Cochrane Preferred Reporting Items for Systematic Reviews and Meta-Analyses (PRISMA) was used to collate data and report results (21). An electronic literature search was performed in MEDLINE (via PubMed), CENTRAL, EMBASE, and ClinicalTrials.gov website from inception to March 14, 2020. Keywords included were as follows: infliximab, etanercept, certolizumab, adalimumab, natalizumab, onercept, vedolizumab, ustekinumab, risankizumab, tofacitinib, filgotinib, fontolizumab, biologic, small targeted molecule, CD, and randomized controlled trial. Comprehensively, generic, code, and trade names of each drug were searched simultaneously. Abstracts from the United European Gastroenterology Week (UEGW), the American College of Gastroenterology (ACG), Digestive Disease Week (DDW), and the Congress of European Crohn’s and Colitis Organisation (ECCO) were searched until 2019. Reference lists of previous reviews were also searched for possible articles. Specific inclusion criteria were listed as follows:

1. Double-blinded randomized clinical trials reported with control treatment.
2. Included patients were at least 18 years old with moderate-to-severe active CD. CD was confirmed by radiologic, endoscopic, or histologic criteria.
3. Patients were treated with biologics or small targeted molecules. Concomitant medications, such as 5-ASA, oral steroids, and immunomodulators (azathioprine, 6-mercaptopurine, or methotrexate), were allowed. History of tumor necrosis factor (TNF) inhibitor was allowed.
4. Trials reported one of the following outcomes: CDAI score, ΔCDAI (change from baseline in CDAI), CDAI150, CDAI-100, CDAI-70, CRP, ΔCRP (change from baseline in CRP), IBDQ, and ΔIBDQ (change from baseline in IBDQ).

Search results were screened, and data were extracted by two reviewers (BY and SJ) independently. Disagreements between two reviewers were resolved by discussion and consensus with a third reviewer (LZ). Only data from trials of the induction period were included. Exclusion criteria included the following: trials in patients having surgery for CD within 3 months, trials without available baseline characteristics, and trials with a combination of anti-TNF and other biologics or small targeted molecules. Data extracted from citations included but were not limited to the following: publication year, title, author, trial name, trial design, and primary outcome. Patient demographics were captured, as well as treatment information of each arm, such as dose, frequency, and administration routes.

Efficacy outcomes were extracted from text, tables, and figures, including CDAI150, CDAI-100, CDAI-70, CDAI, IBDQ, and CRP. Different dose regimens were normalized by daily dose; for example, upadacitinib 12 mg twice daily was standardized to upadacitinib 24 mg daily. Dose regimens that need to be calculated by weight were normalized by 70 kg per patient. The CRP, which was reported in mg/dL or mg/L, was standardized into mg/L. Changes from baseline in continuous outcomes were extracted from articles directly or calculated by subtracting postbaseline values from baseline values. The relative effect was extracted for our analysis to be able to reduce the bias of estimation (15).

In the development of the analytical dataset, intent-to-treat populations were used whenever available. When multiple statistic values were available, the mean value was chosen over the median value. For the trial arms that were stratified by the baseline level, only the outcome values characterizing the overall level of the trial arms were included. The missing values of SDs were imputed by exploring the fixed-effect, linear, log, exponential, and maximum effect (*E*_max_) models. The model-predicted SD values, combined with given SD values, were then used for derivation of weights during the model development. For missing covariates, if the missing values were ≤40%, the median value of the database was used for interpolation, and if the missing values were >40%, the baseline characteristic was not incorporated into the final models.

### Risk of Bias Assessment

The risk of bias was assessed by two investigators independently using the Cochrane risk of bias tool. The evaluation items included random sequence generation, allocation concealment, blinding of participants and personnel, blinding in the outcome assessment, incomplete outcome data, selective reporting, and other biases (22). Disagreements were resolved through discussion with a third investigator.

### Model Development

Data of all the dose regimens were utilized to explore the potential dose–response relationship. However, only data from multiple-dose trials as well as data from the single-dose trial whose time point within the minimum dosing interval of multiple-dose trials were included in the modeling. The longitudinal profiles of efficacy outcomes were characterized using a hierarchical regression model with the maximum likelihood estimation method. To avoid misestimation of placebo effects, a non-parametric method was implemented to estimate placebo effects in each trial and at each time point. The model could be generally described as


(1) $$E_{ijt}=E_{0it}+E_{drug}$$

(2) $$E_{\text{drug}}=f(\text{drug,dose,regimen,time,}\theta {\text{,X}}_{ij})$$

*E_ijt_* represents the efficacy in the *j*th treatment arm of the *i*th trial at *t* time, which is the sum of *E*_0_ *_it_* (the placebo effects of the *i*th trial at *t* time) and *E*_drug_ (the drug effects in the *j*th treatment arm of the *i*th trial at *t* time). For outcomes measured as probability, a logit translation was performed to limit the probability to a range of 0–1. *E*_drug_ is a function dependent on the type of drug, dose, regimen, time, fixed-effect model parameters *θ*, and covariates *X*.

At first, the drug effects were set not to change over time. Then, during model development, if model fit improved, a time variable was added to create a non-linear model to describe the time-varying drug effects. The formula was listed as follows:


(3) $$E_{\text{drug}}=E_{\max_{\text{drug}}}\cdot (1-e^{-k\cdot time})$$

where $E_{max_{drug}}$ represents the maximum efficacy of each treatment and *k* represents the rate constant describing the onset of drug effect.

In the process of the model development, the maximum efficacy of each drug was initially incorporated to be constant over different dosages and described by a scaling factor, *E*_max_. Then, the parameter *E*_max_ was separated into several parameters matching different dose regimens. For drugs with dose range, a dose–response relationship was estimated by *E*_max_ or sigmoid *E*_max_ model. For drugs with poor dose levels, it was hard to estimate a clear dose–response relationship with *E*_max_ model, so a simple fixed-effect or linear dose–response model was used.

Weight was introduced according to the standard error of fitted values for CDAI150, CDAI-100, and CDAI-70 models, and the standard error of observed values for CDAI, CRP, and IBDQ models (Equations 4 and 5). The number of subjects for each trial arm within each trial (N) ensured that more influence on estimating the parameters was imposed by the larger studies.


(4) $$Weight=\frac{SD}{\sqrt{N}}$$

(5) $$Weight=\sqrt{\frac{P\cdot (1-P)}{N}}$$

A more technical exposition is available in the model development section of **Supplementary Materials**.

### Covariate

Baseline characteristics, including age, percentage of male, disease duration, smoking status, CDAI, CRP, and IBDQ, were set as the covariates in the model. Covariates were investigated for their possible impact on the treatment efficacies with the following equation, where *θ* was the parameter quantifying the covariate effect.


(6) $${\text{Effect}}_{\text{Covariate}}=\frac{{\text{Covariate}}^{\theta }}{\text{mean (Covariate)}}$$

Different correlation forms were tested as the within-arm autocorrelation structure, such as AR1, AR2, compound symmetry, and autoregressive moving average structure. Model development and iteration were based on the data and guided by successful convergence of the minimization routine. Model selection was based on the Akaike information criterion and the log-likelihood ratio at an acceptance *p*-value of 0.05.

### Model Evaluation and Simulation

The model fits across trials were evaluated by model-fitted time-course plots and diagnostic plots. The parameters from the final models were used to sample a total of 10,000 model parameters for predicting the treatment efficacies at hypothetical time points.

All data exploration and model development, evaluation, and simulation were carried out with the R software version 3.6.3 [R Core Team (2020)] and the “gnls” function in the “nlme” package version 3.1–145. Literature quality assessment was performed using the Review Manager (RevMan), version 5.4.1, The Cochrane Collaboration, 2020.

## Results

### Available Data

**Figure 1:**
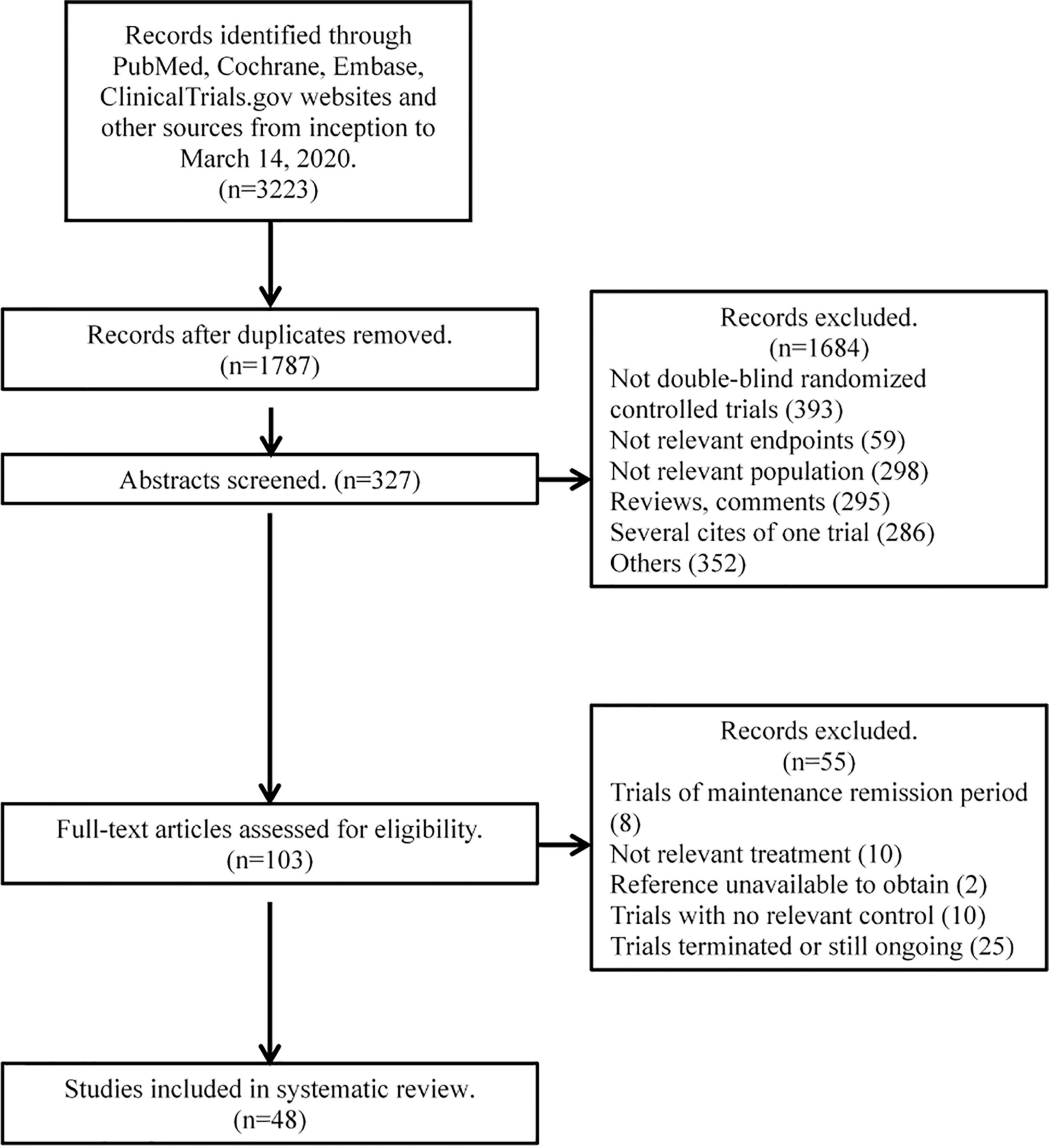
Flow diagram for study selection.

A total of 3,223 citations were retrieved from the initial search. After the review of the abstracts and full articles, 46 trials containing 146 treatment arms and 12,846 patients were included in the analysis (12–14, 23–64). The complete process of literature searching and screening is shown in the flow diagram (**Figure 1**). Among the 46 included trials, the overall quality was assessed as high with a low risk of bias; detailed information on the assessment of literature quality is shown in **Supplementary Figures 1** and **2**.

Seventeen biologics and 7 small targeted molecules were involved, including TNF-α inhibitors, integrin inhibitors, interleukin (IL) inhibitors, matrix metalloproteinase-9 (MMP-9) inhibitor, janus kinase (JAK) inhibitors, T-cell activation inhibitors, mucosal addressing cell adhesion molecule (MAdCAM) inhibitor, interferon (IFN)-γ inhibitor, and C-C chemokine receptor-9 (CCR-9) antagonist. The drug classification and overview of included trials as well as prespecified baseline characteristics are displayed in **Table 1**.

**Table 1:** Summary of available information for each drug in the analysis.

Drug	Trials	Patients	Arms	Route (regimen)	Percentage of male (%)	Age (years)	Disease duration (years)	Baseline CDAI	Baseline CRP	Baseline IBDQ
**TNF-α inhibitor**										
Infliximab	3	274	7	i.v. (5, 10, 20 mg/kg) i.v. (5 mg/kg 0, 2, 6, q8w)	49.94	34.82	5.59	298.84	1.55	123.10
CDP571	2	284	2	i.v. (5, 10 mg/kg)	40.28	38.17	9.20	290.78	0.67	129.00
Etanercept	1	23	1	s.c. (25 mg biw)	69.60	37.40	NA	299.50	NA	124.40
Certolizumab pegol	5	899	10	i.v. (5, 10, 20 mg/kg) s.c. (200, 400 mg q2w) s.c. (100, 200, 400 mg q4w) s.c. (400 mg 0, 2, 4, q4w)	47.07	36.31	7.59	290.53	0.89	126.87
Adalimumab	4	553	7	s.c. (40, 80, 160 mg 0w followed by 20, 40, 80 mg 2w) s.c. (160 mg 0w followed by 80 mg 2w followed by 40 mg 4, 6w)	54.14	34.85	10.73	294.38	1.73	136.81
Onercept	1	169	4	s.c. (10, 25, 35, 50 mg tiw)	42.01	36.06	9.91	316.51	2.09	NA
Semapimod	1	97	2	i.v. (60 mg qd for 1, 3d)	46.36	37.47	9.68	320.01	2.94	121.53
**Integrin-α4 inhibitor**										
Natalizumab	4	1,186	6	i.v. (3 mg/kg) i.v. (3, 6 mg/kg q4w) i.v. (300 mg q4w)	42.70	37.53	9.77	300.66	2.07	125.53
**Integrin-α4β7 inhibitor**										
Vedolizumab	4	635	5	i.v. (0.5, 2 mg/kg q4w) i.v. (300 mg 0, 2w) i.v. (300 mg 0, 2, 6w)	47.41	36.20	8.78	317.25	2.66	131.00
Abrilumab	1	154	3	s.c. (21, 70, 210 mg 0, 1, 2, q4w)	44.16	36.42	11.19	314.74	NA	NA
**IL-12/23 inhibitor**										
Ustekinumab	4	1,357	9	i.v. (1, 3, 4.5, 6 mg/kg) i.v. (130 mg) s.c. (90 mg qw)	45.91	38.29	11.18	318.57	1.02	NA
Apilimod	1	147	2	p.o. (50, 100 mg qd)	38.78	41.00	11.09	301.99	NA	NA
**IL-23 inhibitor**										
Risankizumab	1	82	2	i.v. (200, 600 mg q4w)	62.20	39.35	14.00	304.06	0.95	NA
Brazikumab	1	59	1	i.v. (700 mg q4w)	37.29	34.90	13.10	325.00	2.98	NA
**IL-6 inhibitor**										
PF-04236921	1	179	3	s.c. (10, 50, 200 mg q4w)	42.17	39.64	10.64	314.61	2.18	NA
**MMP-9 inhibitor**										
Andecaliximab	1	159	3	s.c. (150 mg q2w) s.c. (150, 300 mg qw)	52.83	39.67	12.23	328.00	2.11	NA
**JAK inhibitor**										
Tofacitinib	2	293	5	p.o. (1, 5, 10, 15 mg bid)	54.15	39.29	11.18	311.63	1.11	NA
Upadacitinib	1	183	5	p.o. (3, 6, 12, 24 mg bid) p.o. (24 mg qd)	44.81	40.72	10.69	289.23	0.95	NA
Filgotinib	1	130	1	p.o. (200 mg qd)	45.38	37.40	8.80	291.30	1.42	NA
**T-cell activation inhibitor**										
Laquinimod	1	117	4	p.o. (0.5, 1, 1.5, 2 mg qd)	39.31	39.02	NA	297.75	1.13	NA
Abatacept	1	323	3	i.v. (3, 10 mg/kg 0, 2, 4, 10w) i.v. (30 mg/kg 0, 2w followed by 10 mg/kg 4, 10w)	39.94	37.39	9.32	318.84	2.49	NA
**MAdCAM inhibitor**										
Ontamalimab	1	199	3	s.c. (22.5, 75, 225 mg q4w)	36.68	35.87	12.04	315.93	1.76	NA
**IFN-γ inhibitor**										
Fontolizumab	2	251	6	i.v. (4, 10 mg/kg q4w) i.v.–s.c. (1, 4 mg/kg 0w followed by 0.1, 1 mg/kg q4w)	45.42	36.51	8.61	315.35	2.11	125.51
**CCR-9 receptor blocker**										
Vercirnon	2	696	5	p.o. (250, 500 mg qd) p.o. (250, 500 mg bid)	44.22	36.46	8.45	323.97	2.02	NA
**Placebo**	46	4,397	47		46.79	37.07	9.14	304.18	1.71	126.66
**Total**	46	12,846	146		46.09	37.20	9.52	306.75	1.67	127.37

CDAI, Crohn’s Disease Activity Index; CRP, C-reactive protein; IBDQ, Inflammatory Bowel Disease Questionnaire; NA, not available.

Most reported outcomes were CDAI150, CDAI-100, CDAI-70, ΔCDAI, ΔCRP, and ΔIBDQ, which were evaluated in 38, 27, 24, 21, 26, and 20 trials, respectively. These six outcomes were selected for modeling. Among them, CDAI150 was defined as clinical remission, while CDAI-100 or CDAI-70 were defined as the clinical response. The detailed information about reported time points of each outcome in included trials was shown in **Supplementary Table 1**.

Before modeling, all the prespecified covariates were screened, and missing values of age, percentage of male, disease duration, smoking status baseline CDAI, CRP, and IBDQ were found in 0%–38% of the trials. To further develop the database, missing values were imputed with the median values of given baseline characteristics. The database used for the MBMA can be found in **Supplementary Materials**.

### Final Models

The time course and dose–response relationship were adequately described by the longitudinal models, which were shown as follows:


(7) $$E_{\text{drug}}=E_{{\text{max}}_{\text{drug}}}\cdot (1-e^{-k\cdot time})$$

(8) $$E_{{\text{max}}_{\text{drug}}}=f(\text{drug,dose,regimen})$$

**Figure 2:**
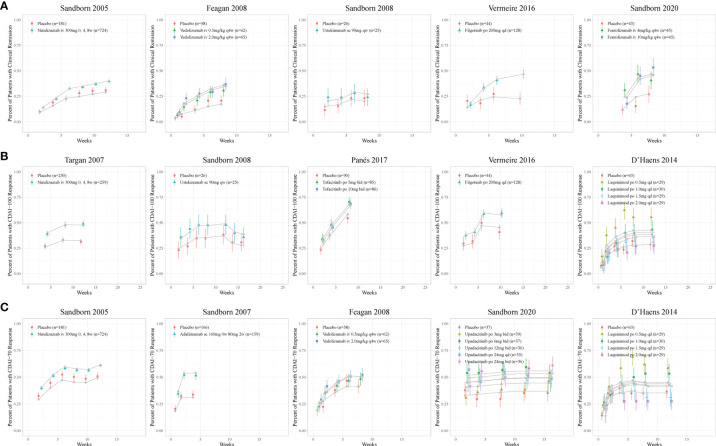
Model fitted time-course plots of response rate for **(A)** CDAI150, **(B)** CDAI-100, and **(C)** CDAI-70 for representative trials. Color symbols and vertical bars are observed mean and calculated weight of time points; gray symbols and lines are the model predictions. CDAI, Crohn’s Disease Activity Index; CDAI150, an absolute CDAI score of less than 150; CDAI-100, reduction of at least 100 points in the CDAI score; CDAI-70, reduction of at least 70 points in the CDAI score; qd, once daily; bid, twice daily; qw, once weekly; q4w, once every 4 weeks.

**Figure 3:**
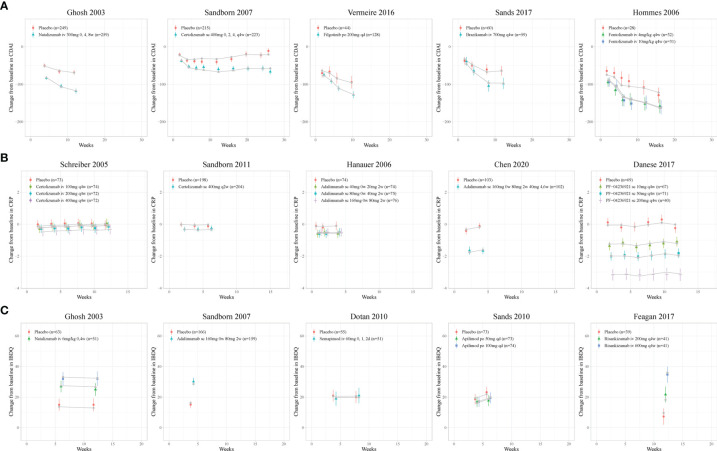
Model fitted time-course plots of **(A)** CDAI, **(B)** CRP, and **(C)** IBDQ change from baseline for representative trials. Color symbols and vertical bars are observed mean and calculated weight of time points; gray symbols and lines are the model predictions. CDAI, Crohn’s Disease Activity Index; CRP, C-reactive protein; IBDQ, Inflammatory Bowel Disease Questionnaire; qd, once every day; q2w, once every 2 weeks; q4w, once every 4 weeks.

The model fitted time-course plots of representative trials for six models are shown in **Figures 2** and **3**, and additional plots can be found in **Supplementary Materials**. The time-varying drug efficacy was found in the CDAI150 and ΔCDAI models, as the exponential function shown in Equation 7, where $E_{max_{drug}}$ represents the maximum efficacy, and *k* represents the rate constant describing the onset of drugs. Based on the exponential model (**Figure 2**), the time to reach 50% of the maximum effect (ET_50_) of JAK inhibitors was estimated to be about 6.3 weeks, and the time to reach 90% of the maximum effect (ET_90_) was estimated to be 20.9 weeks. Moreover, *k_general_* (the rate constant for all treatments) was estimated for the ΔCDAI model. Based on the estimated values, ET_50_ and ET_90_ were assumed to be 3.2 and 10.5 weeks in the ΔCDAI model. Dose–response relationship was estimated for each drug with *E*_max_ model. For drugs without enough information available in the database to estimate a clear dose–response relationship, a simple fixed-effect or linear model was used.

## Covariates

Seven prespecified covariates (percentage of men, age, disease duration, smoking status, CDAI, CRP, and IBDQ) were tested for their association with the drug efficacies. Age, disease duration, baseline CDAI, and CRP were included in the final models. For CDAI150, CDAI-100, CDAI-70, and ΔCDAI model, the estimated covariate parameters of negative value for baseline CDAI (**Table 2**) indicated that the patients with lower baseline CDAI were expected to get greater efficacy. The parameters for age (−7.69 [95% CI: −11.11 to −4.26]) in the CRP model means that younger patients were assumed to get more decrease in CRP. Baseline CRP was also estimated as a covariate in the CDAI150, CDAI-100, CDAI-70, and ΔCRP model (**Table 2**), which means that patients with higher baseline CRP are assumed to get more improvement measured in CDAI and less decrease in CRP. The covariate parameters for disease duration were estimated as 4.95 (95% CI: 3.60 to 6.29) for ΔCRP and −8.98 (95% CI: −10.60 to −7.36) for ΔIBDQ, indicating a better decrease in CRP and less improvement in IBDQ among patients with longer CD duration.

**Table 2:** Estimate of key parameters in final models.

Model	Parameter		Estimate	95%CI
CDAI150 model	*E_drug_*	Adalimumab (slope)^a^	6.00×10^-3^	(4.22×10^-3^, 7.78×10^-3^)
		Risankizumab (slope)^a^	2.55×10^-3^	(8.88×10^-4^, 4.22×10^-3^)
		PF-04236921 (slope)^a^	8.33×10^-3^	(2.92×10^-3^, 1.37×10^-2^)
	*k_JAK_*	Rate constant for the onset of JAK inhibitor	0.11	(0.01, 1.40)
	Covariate	Baseline CDAI	-5.22	(-7.90, -2.54)
		Baseline CRP	0.51	(0.20, 0.83)
CDAI-100 model	*E_drug_*	Adalimumab (Emax)^a^	0.93	(0.30, 1.56)
		Adalimumab (ED50)^a^	34.13	(1.66, 703.51)
		Upadacitinib (slope)^b^	4.34×10^-2^	(6.32×10^-3^, 8.04×10^-2^)
	Covariate	Baseline CDAI	-8.77	(-14.16, -3.39)
		Baseline CRP	0.29	(-0.14, 0.71)
CDAI-70 model	*E_drug_*	Upadacitinib (Emax)^a^	0.89	(0.09, 1.69)
		Upadacitinib (ED50)^a^	3.91	(0.19, 79.30)
	Covariate	Baseline CDAI	-2.03	(-5.37, 1.32)
		Baseline CRP	0.27	(-0.12, 0.67)
ΔCDAI model	*E_drug_*	Adalimumab (Emax)^a^	-151.24	(-322.11, 19.64)
		Adalimumab (ED50)^a^	112.23	(9.02, 1395.85)
	*k_general_*	Rate constant for the onset of all drugs	0.22	(0.14, 0.34)
	Covariate	Baseline CDAI	-2.05	(-4.93, 0.83)
ΔCRP model	*E_drug_*	Certolizumab pegol (slope)^b^	1.12×10^-2^	(-1.70×10^-2^, -5.47×10^-4^)
		PF-04236921 (Emax)^a^	-7.47	(-12.76, -2.18)
		PF-04236921 (ED50)^a^	93.69	(41.26, 241.86)
		Upadacitinib (slope)^b^	-0.22	(-0.43, -0.01)
	Covariate	Age	-7.69	(-11.11, -4.26)
		Disease duration	4.95	(3.60, 6.29)
		Baseline CRP	-0.87	(-1.24, -0.50)
ΔIBDQ model	*E_drug_*	Adalimumab (slope)^a^	0.10	(0.05, 0.15)
		Risankizumab (slope)^a^	0.05	(0.01, 0.10)
	Covariate	Disease duration	-8.98	(-10.60, -7.36)

CDAI150, an absolute CDAI score of less than 150; CDAI-70, reduction of at least 70 points in the CDAI score; CDAI-100, a reduction of at least 100 points in the CDAI score; ΔCDAI, change form baseline in CDAI; CDAI, Crohn’s Disease Activity Index; ΔCRP, change form baseline in CRP; CRP, C-reactive protein; ΔIBDQ, change form baseline in IBDQ; IBDQ, inflammatory bowel disease questionnaire; 95% CI, 95% confidence interval; Emax, maximum drug efficacy; NA, not available; qd, once daily; bid, twice daily; qw, once weekly; q4w, once every 4 weeks; q8w, once every 8 weeks.

a Emax model with a E_max_ and a ED_50_ was used for the dose-response relationship.

b Linear model with a slope was used for the dose-response relationship.

More detailed results and code were available in the final model section of **Supplementary Materials**.

### Model Simulation

To compare all treatments, drug effect at week 12, the most common duration of the induction period, as well as the most common time point of primary outcome among included trials, was simulated with final models. A typical trial indicating the common characteristic of included trials was assumed for the simulation with a hypothesis population with 46.09% men, 9.52 years of disease duration, baseline CDAI of 306.75, and baseline CRP of 1.67. To generate the simulation, a longitudinal placebo model was developed for each outcome.

**Figure 4:**
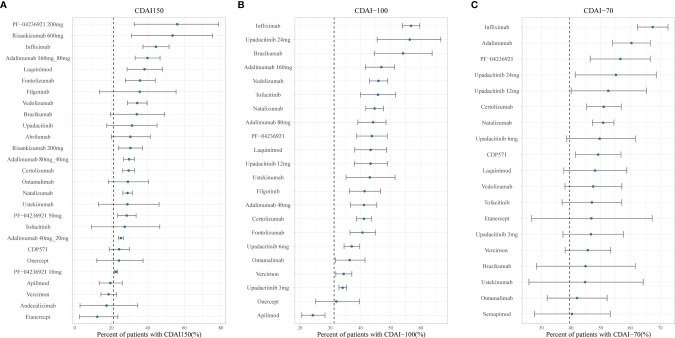
Ranking of the treatments by predicted placebo-corrected median percent of patients with **(A)** CDAI150, **(B)** CDAI-100, and **(C)** CDAI-70 at week 12 (from high to low). Point estimates and 95% CIs were predicted from a model simulation of N = 10,000. Dashed lines represent simulated placebo efficacy. For treatments with multiple dosage regimens, only regimens with different efficacy at week 12 were listed separately. CDAI, Crohn’s Disease Activity Index; CDAI150, an absolute CDAI score of less than 150; CDAI-100, reduction of at least 100 points in the CDAI score; CDAI-70, reduction of at least 70 points in the CDAI score; bid, twice daily.

**Figure 5:**
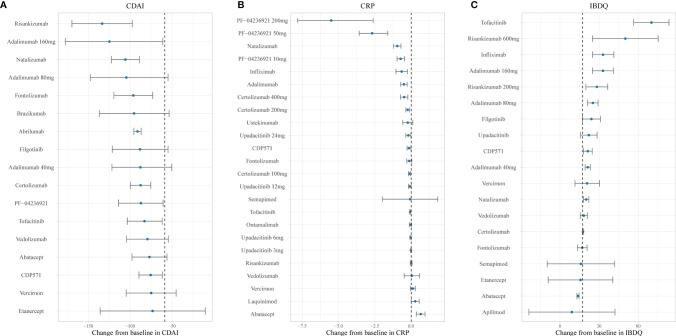
Ranking of the treatments by predicted placebo-corrected median change in **(A)** CDAI, **(B)** CRP, and **(C)** IBDQ at week 12 (from high to low). Point estimates and 95% CIs were predicted from a model simulation of N = 10,000. Dashed lines represent simulated placebo efficacy. For treatments with multiple dosage regimens, only regimens with different efficacy at week 12 were listed separately. CDAI, Crohn’s Disease Activity Index; CRP, C-reactive protein; IBDQ, Inflammatory Bowel Disease Questionnaire.

**Figures 4** and **5** show the ranking of median placebo-corrected treatment effects at 12 weeks for each outcome. Among the result of the simulation for the six outcomes, the consistent highest efficacy was provided by the TNF-α inhibitor, IL-23 inhibitor, and integrin-a4 inhibitor with a narrow 95% CI. The model simulation of the CDAI150, with a placebo effect estimated as 21.26%, is shown in **Figure 4A**. It reveals that PF-04236921 200 mg had the best response in CDAI150 (median: 56.12%, 95% CI: 32.55% to 78.83%) with a large 95% CI, followed by risankizumab 600 mg (median: 53.55%, 95% CI: 31.09% to 75.39%) and infliximab (median: 44.44%, 95% CI: 37.39% to 51.63%). For CDAI-100 (**Figure 4B**), with a placebo effect estimated as 31.28%, infliximab was predicted to have the highest efficacy as 56.75% (95% CI: 51.12% to 62.37%), followed by upadacitinib (median: 56.29%, 95% CI: 35.41% to 76.68%) and brazikumab (median: 54.13%, 95% CI: 35.39% to 72.86%) with a large 95% CI. For the response rate of CDAI-70 (**Figure 4C**), the placebo effect was estimated as 39.48%. Infliximab (median: 67.49%, 95% CI: 62.33% to 72.63%) and adalimumab (median: 60.34%, 95% CI: 54.02% to 66.65%) were predicted to have the highest drug efficacy. As presented in **Figure 5A**, the most placebo-corrected decrease in CDAI was simulated for risankizumab (median: −133.40, 95% CI: −169.53 to −97.35) and adalimumab 160 mg (median −124.75, 95% CI: −177.58 to −60.98) with large 95% CI. The placebo effect was estimated as −58.67. For CRP, the placebo effect was simulated as 0.016 with a longitudinal placebo model. PF-04236921 200 mg (median: −5.52, 95% CI: −7.83 to −2.63) was shown to be the most effective regimen with a large 95% CI. Natalizumab (median: −0.95, 95% CI: −1.21 to −0.70) also showed great efficacy. Ranking of the treatments by predicted IBDQ improvement (**Figure 5C**) showed that tofacitinib (median: 70.01, 95% CI: 56.56 to 83.48) was predicted to be most effective. The placebo effect was simulated as 17.36 with a longitudinal model, shown as the dashed line in **Figure 5C**.

### Residual Correlation

After comparison of the model fit, the AR1 model was used to account for residual correlation for the CDAI-70, ΔCDAI, and ΔIBDQ models; AR2 model was used for CDAI150 and ΔCRP models; and compound symmetry structure was used for the CDAI-100 model.

## Discussion

Our MBMA quantitatively compared the efficacy of 24 drugs in six outcomes. To normalize the comparison, the analysis was adjusted by the percentage of male patients, duration of disease, age, smoking status, baseline CDAI, baseline CRP, baseline IBDQ, dose regimen, and administration route. The estimated model was used to predict and compare drug effects, which could help physicians make appropriate treatment strategies. For example, **Figure 4** demonstrates that infliximab shows the best efficacy in clinical response. These results were known from trials (65, 66) or meta-analyses (2, 10, 11, 67–69); however, most of those researches focused on only one or several specific classes of biologics. Our study is the first to simultaneously evaluate and report all the biologics and small targeted molecules with a quantitative method, which quantified the efficacy of each drug as well as the influence of dosage, time, and covariates.

A total of six outcomes, including continuous outcomes (ΔCDAI, ΔCRP, and ΔIBDQ) and binary outcomes (CDAI150, CDAI-100, and CDAI-70), were evaluated. They mainly assessed the improvement in disease activity and life quality, as well as change of biomarker. To provide a comprehensive understanding, drug efficacies were evaluated based on these three aspects. CDAI was the most commonly used scoring system in clinical practice. However, some clear limitations were observed in it (e.g., interobserver variability) (8, 70), so objective laboratory test data (CRP) and scoring system for life quality (IBDQ) (19, 70) were also included in our analysis. Although CDAI150, CDAI-100, and CDAI-70 are determined by the same CDAI score, they were reported in different trials. To catch the whole picture of targeted treatment in CD, all these outcomes were included in the analysis. Mucosal healing is considered a therapeutic goal of CD (4, 71); however, most of the included trials did not report consistent endoscopic outcomes, and the efficacy of achieving endoscopic remission was not examined in our analysis. Some of the outcomes and covariates were not reported in all included trials (46); however, as the missing value is considered random, the result would be considered unbiased (72).

In general, the efficacy trends of drugs measured in six outcomes were similar. TNF-α inhibitors were the most effective biologics, followed by integrin inhibitors and IL-12/23 inhibitors. For small targeted molecules, the highest efficacy was observed in JAK inhibitors. These results were supported by several previous meta-analyses (2, 10, 11, 67–69). However, there were still some differences in the rank order across six outcomes between our results and previous research, which was attributed to the different covariate effects estimated for each outcome, and the limit in sample size. The results should be interpreted with caution.

The onset speed of drug effects in CD is of great importance for physicians to adjust treatment plans in time. In our longitudinal MBMA, the time-course models could quantitatively estimate the onset of drugs. However, time-response relationships were only found in CDAI150 and ΔCDAI, which was reported by most trials. Although it is generally accepted that continuous outcomes were more sensitive to changes (73), there may not be enough data to estimate a time-course model for CRP and IBDQ. Because of the long T_1/2_ of monoclonal antibodies, which is reported to be 15–51 days or more (25, 74), it may be unable for some drugs to reach steady-state during the induction period of remission, which may also be the reason for the poor result in the estimation of time-response relationships. Therefore, caution is needed in interpreting the results.

The impact of dose regimen on the treatment efficacy was also tested in our analysis. The dose–response relationships of adalimumab, upadacitinib, certolizumab pegol, risankizumab, and PF-04236921 were identified by *E*_max_ or linear model, indicating that a higher dose could improve the drug efficacy. A common *E*_max_ parameter was tried to be estimated during the modeling. However, due to the poor sample size or lack of dose range, the dose-varying efficacy was only successfully estimated in several drugs. It may also result from short research duration (15), as more visible dose–response relationships were observed in longer-term efficacy in trials (14, 26).

A benefit of MBMA is that the impact of covariates on the treatment efficacy can be quantitatively described (75), and five covariates were included in our final models. Patients with lower baseline CDAI were shown to have greater improvement in CDAI150, CDAI-100, CDAI-70, ΔCDAI, and ΔCRP, which was consistent with previous studies (75). In addition, a lower CRP level was also considered as a predictor of more decrease in CRP (76). Our analysis suggested that younger patients were shown to get better efficacy in ΔCRP models. In the ΔIBDQ model, patients with shorter disease duration showed more improvement, while more response in the ΔCRP model was shown in patients with longer disease duration. In other studies, early intervention with biologics was considered to show more benefits (77). The reason for the difference may be that early intervention was defined as 8 weeks to 2 years in other studies (77), but in our analysis, the mean disease duration of included trials was 9.5 years. The study of CD’s natural history suggested that a longer course of the disease may lead to more serious and complex conditions (78), so there may be a correlation between the duration and severity of the disease. Moreover, our identification of covariates was based on the aggregation of trial-level data, which contains less information than patient-level data (79, 80). Besides, for models with few baseline characteristics available, there may not be enough power to detect the impact of covariates on drug efficacy.

There are several advantages of our MBMA. First, our analysis included the largest number of trials, drugs, and patients. Second, longitudinal models and different dose–response models were used to describe the drug effect. Thus, data of drugs in different dose regimens at all the time points were able to be utilized in the analysis. Third, our inclusion criteria limited the studies to randomized controlled trials (RCTs), reducing unnecessary biases between treatment arms. Besides, the placebo effect for each trial was estimated respectively, because of the non-negligible between-trial variability among placebo effects of trials for CD (81). Thus, we were able to quantify the relative drug effect in an unbiased way. Fourth, the framework can be adapted and reused in other drugs for CD, and the model can be easily updated with more data of clinical efficacy available.

There are still some limitations in our analysis. First, it should be noticed that heterogeneity in the population was observed in several trials. For example, trials of some drugs included patients who had no response to previous treatment (46). This may lead to lower efficacy for these drugs in our analysis. However, previous studies indicated that prior exposure to both anti-TNF and other treatments did not impact the result of comparison between biologics significantly (2, 10, 69, 82). Moreover, the combined efficacy of anti-TNF-naive and anti-TNF-exposed patients was reported in several trials, so it is difficult to estimate the impact of anti-TNF experience separately. Second, the generalizability of our results is limited to the population enrolled in the included trials. For example, the inclusion criteria limited patients in moderate-to-severe CD, and thus, our interpretations do not necessarily relate to patients with mild disease activity. Third, mucosal healing, which is considered an important therapeutic endpoint in the management of CD, was not included in our meta-analysis due to the deficiency in data. Fourth, efficacy data were still inadequate for some drugs, which may lead to imprecise and unreliable estimates (15). Thus, caution is needed in interpreting these results.

In conclusion, our analysis provided an MBMA framework that combined evidence from 46 RCTs, allowing the estimate and prediction of efficacy for multiple agents across time course and a range of doses. In general, TNF-α inhibitors were the most effective biologics, and the highest efficacy of small targeted molecules was observed in JAK inhibitors. Besides, the patients’ age, disease duration, baseline CDAI, and CRP were identified as the covariates that show the impact on drug efficacy. We hope that our results will enable physicians and patients to understand better the differences and similarities across 17 biologics and 7 small targeted molecules in CD for 6 important outcomes.

## Data Availability Statements

The original contributions presented in the study are included in the article/**Supplementary Material**. Further inquiries can be directed to the corresponding authors.

## Author Contributions

XW, JZ, BY, LZ, SJ, and HH wrote the manuscript. XW, JZ, BY, and LZ designed the research. BY, LZ, SJ, and HH performed the research. BY, LZ, SJ, and HH analyzed the data. All authors listed have made a substantial, direct, and intellectual contribution to the work and approved it for publication.

## Funding

This work was supported by the National Science and Technology Major Project [Grant number 2018ZX09721003] and Beijing Municipal Natural Science Foundation [Grant number L202042].

## Conflict of Interest

The authors declare that the research was conducted in the absence of any commercial or financial relationships that could be construed as a potential conflict of interest.

## Publisher’s Note

All claims expressed in this article are solely those of the authors and do not necessarily represent those of their affiliated organizations, or those of the publisher, the editors and the reviewers. Any product that may be evaluated in this article, or claim that may be made by its manufacturer, is not guaranteed or endorsed by the publisher.
