# Supplementary material for: Model-Based Meta-Analysis on the Efficacy of Biologics and Small Targeted Molecules for Crohn’s Disease
Source: Front Immunol. 2022 Mar 17;13:828219. doi: 10.3389/fimmu.2022.828219 (PMC8967940; doi:10.3389/fimmu.2022.828219)
Supplement: Supplementary file 2 [file DataSheet_2.pdf]

Supplementary Materials

Model-Based Meta-Analysis on the Efficacy of Biologics and Small Targeted Molecules for Crohn's Disease

Table of Contents

Time Points of Included Studies 2

Risk of Bias Assessment..7

Model Development 9

Final Models 12

Parameters and model fitted time-course plots CDAI150 model 12

Parameters and model fitted time-course plots CDAI-100 model..16

Parameters and model fitted time-course plots CDAI-70 model...18

Parameters and model fitted time-course plots ΔCDAI model...20

Parameters and model fitted time-course plots ΔCRP model...22

Parameters and model fitted time-course plots ΔIBDQ model...25

Diagnostic Plots 27

Model Code...29

Code for CDAI150 Model 29

Code for CDAI-100 Model..32

Code for CDAI-70 Model..35

Code for ΔCDAI Model...38

Code for ΔCRP Model...41

Code for ΔIBDQ Model...44

References and Time Points of Included Studies

***Supplementary Table* 1** Time points for studies included in the model-based meta-analysis.

No.	Study	Patients	Drug	Regimen	Time points (week)					
					CDAI	CDAI70	CDAI100	CDAI150	IBDQ	CRP
1	Targan, 1997	108	infliximab	5 mg/kg<br>10 mg/kg<br>20 mg/kg	2,4	2,4,12		2,4,12	4	2,4
2	D'haens 1999	30	infliximab	5 mg/kg<br>10 mg/kg<br>20 mg/kg	4					4
3	Colombel, 2010 (SONIC)	339	infliximab	5 mg/kg 0,2,6 q8w		2,6,10,18, 26	2,6,10,18, 26	2,6,10,18, 26	2,6,10,18, 26	26
4	Gordon, 2001	30	natalizumab	3 mg/kg	1,2,4			2		
5	Ghosh, 2003	248	natalizumab	3 mg/kg 0w<br>3 mg/kg 0,4w<br>6 mg/kg 0,4w		2,4,6,8,12		2,4,6,8,12	6,12	
6	Sandborn, 2005 (ENACT-1)	905	natalizumab	300 mg 0,4,8w		2,4,6,8,10 ,12		2,4,6,8,10 ,12		
7	Targan, 2007 (ENCORE)	509	natalizumab	300 mg 0,4,8w	4,8,12	4,8,12	4,8,12	4,8,12	12	4,8,12
8	Stack, 1997	31	CDP571	5 mg/kg	2,4,6,8			2		2,4,6,8
9	Sandborn, 2004	395	CDP571	10 mg/kg q8w	2,4,8,12,1 6,24,28	2,4,8,12,1 6,24,28		2,4,8,12,1 6,24,28	2,4,8,16,2 4,28	4,8,16,28
10	Sandborn, 2001	43	etanercept	25 mg biw	2,4,8	2,4,8		2,4,8	2,4,8	
11	Winter, 2004	90	certolizumab	5 mg/kg<br>10 mg/kg<br>20 mg/kg	2,4,8,12			2,4,8,12		2,4,8,12
12	C87037	94	certolizumab	200 mg q2w<br>400 mg q2w		2,4,6	2,4,6	2,4,6		
13	Schreiber, 2005	291	certolizumab	100 mg q4w<br>200 mg q4w<br>100 mg q4w				2,4,6,8,10 ,12		2,4,6,8,10 ,12

14	Sandborn, 2011	439	certolizumab	400 mg q2w	2,4,6		2,4,6	2,4,6		2,4,6
15	Sandborn, 2007 (PRESISE-1)	660	certolizumab	400 mg 0,2,4 q4w	2,4,6,8,12<br>,16,20,24,<br>26	2,4,6,8,12<br>,16,20,24,<br>26	2,4,6,8,12<br>,16,20,24,<br>26	2,4,6,8,12<br>,16,20,24,<br>26	26	2,4,6,8,12<br>,16,20,24,<br>26
16	Hanauer, 2006 (CLASS-I)	299	adalimumab	40 mg 0w+20 mg 2w<br>80 mg 0w+40 mg 2w<br>160 mg 0w+80 mg 2w	1,2,4	1,2,4	1,2,4	1,2,4	1,2,4	1,2,4
17	Watanabe, 2011	90	adalimumab	80 mg 0w+40 mg 2w<br>160 mg 0w+80 mg 2w	2,4	4	4	2,4		
18	Sandborn, 2007 (GAIN)	325	adalimumab	160 mg 0w+80 mg 2w	1,2,4	1,2,4	1,2,4	1,2,4	4	4
19	Chen, 2020	205	adalimumab	160 mg 0w+80 mg 2w	2,4	2,4		2,4		2,4
20	Rutgeerts, 2006	207	onercept	10 mg tiw<br>25 mg tiw<br>35 mg tiw<br>50 mg tiw			8	8		
21	Feagan, 2008	185	vedolizumab	0.5 mg/kg q4w<br>2 mg/kg q4w	8	1,2,4,6,8	8,16	1,2,4,6,8	8	
22	Sandborn, 2013 (GEMINI2)	368	vedolizumab	300 mg 0,2w			6	6		6
23	Sands, 2014 (GEMINI3)	416	vedolizumab	300 mg 0,2,6w			6,10	6,10		
24	Watanabe 2019		vedolizumab	300 mg 0,2,6w			10	10		2,6,10
25	Sandborn, 2008	104	ustekinumab	90 mg qw<br>4.5mg/kg		2,4,6,8,12<br>,14,16	2,4,6,8,12<br>,14,16	2,4,6,8,12<br>,14,16		8
26	Sandborn, 2012	526	ustekinumab	1 mg/kg<br>3 mg/kg<br>6 mg/kg	4,6,8	4,6,8	4,6,8	4,6,8	6	4,6,8
27	Feangan, 2016 (UNITI1)	741	ustekinumab	130 mg<br>6 mg/kg	3,6,8	3,6,8	3,6,8	3,6,8	8	3,6,8
28	Feangan, 2016 (UNITI2)	628	ustekinumab	130 mg<br>6 mg/kg	3,6,8	3,6,8	3,6,8	3,6,8		3,6,8

29	Dotan, 2010	152	semapimod	60 mg qd 3d<br>60 mg qd 1d		4,8			4,8	4,8
30	Sands, 2010	701	apilimod	50 mg qd<br>100 mg qd			4,6,24	4,6,24	4,6	
31	Schreiber,<br>2017	187	andecaliximab	150 mg q2w<br>150 mg qw<br>300mg_qw				8		
32	Sandborn,<br>2014	139	tofacitinib	1 mg bid<br>5 mg bid<br>15 mg bid	1,2,4,8	1,2,4	4	4	4	1,2,4,8
33	Panes, 2017	280	tofacitinib	5 mg bid<br>10 mg bid	2,4,8	2,4,8	2,4,8	2,4,8	8	8
34	Vermerire,<br>2016<br>(FITZROY)	174	filgotinib	200 mg qd	2,4,6,10		2,4,6,10	2,4,6,10	10	
35	Sandborn,<br>2012	451	abatacept	3 mg/kg 0,2,4,10w<br>10 mg/kg 0,2,4,10w<br>30 mg/kg 0,2w+10 mg/kg<br>4,10w	2,4,8,12				12	2,4,8,12
36	Feagan,<br>2017	121	risankizumab	200 mg q4w<br>600 mg q4w	4,8,12			4,8,12	12	4,8,12
37	Danese,<br>2017<br>(ANDANTE<br>1)	249	PF-04236921	10 mg q4w<br>50 mg q4w<br>200 mg q4w	2,4,6,8,10<br>,12	2,4,6,8,10<br>,12	2,4,6,8,10<br>,12	2,4,6,8,10<br>,12		2,4,6,8,10<br>,12
38	Sandborn,<br>2018<br>(OPERA)	265	ontamalimab	22.5 mg q4w<br>75 mg q4w<br>225 mg q4w		2,4,6,8,10<br>,12	2,4,6,8,10<br>,12	8,12		4,8,12
39	Sands, 2017	119	brazikumab	700 mg 0,4w	2,4,8,12	8	8	8,12		
40	Sandborn,<br>2020<br>(ELEST)	220	upadacitinib	3 mg bid<br>6 mg bid<br>12 mg bid<br>24 mg bid<br>24 mg qd		2,4,8,12,1<br>6	16	2,4,8,12,1<br>6	8,16	16
41	Hommes,<br>2006	133	fontolizumab	4 mg/kg q4w<br>10 mg/kg q4w	2,4,6,8,12<br>,16		4,6,8	4,6,8	4,6,8,12,1<br>6,28	2,4,6,8,12<br>,16
42	Reinish,<br>2010	201	fontolizumab	1 mg/kg 0w+0.1 mg/kg<br>q4w<br>1 mg/kg 0w+1 mg/kg q4w	4,6,8,12,		4,6,8,12	4,6,8,12		4,6,8,12

				4 mg/kg 0w+0.1 mg/kg q4w<br>4 mg/kg 0w+1 mg/kg q4w						
43	Sandborn, 2017	78	abrilumab	21 mg 1,2,3,q4w<br>70 mg 1,2,3,q4w<br>210 mg 1,2,3,q4w	8,12			8,12		
44	Keshav, 2013 (PROTECT-1)	436	vercirnon	250 mg qd<br>250 mg bid<br>500 mg qd	12	4,8,12	4,8,12	4,8,12		12
45	Feagan, 2015 (SHIELD-1)	608	vercirnon	500 mg qd<br>500 mg bid			8,12	8,12	8,12	12
46	D'Haens, 2014	180	laquinimod	0.5 mg qd<br>1 mg qd<br>1.5 mg qd<br>2 mg qd		1,2,4,6,8, 12	1,2,4,6,8, 12	1,2,4,6,8, 12		8

Risk of Bias Assessment

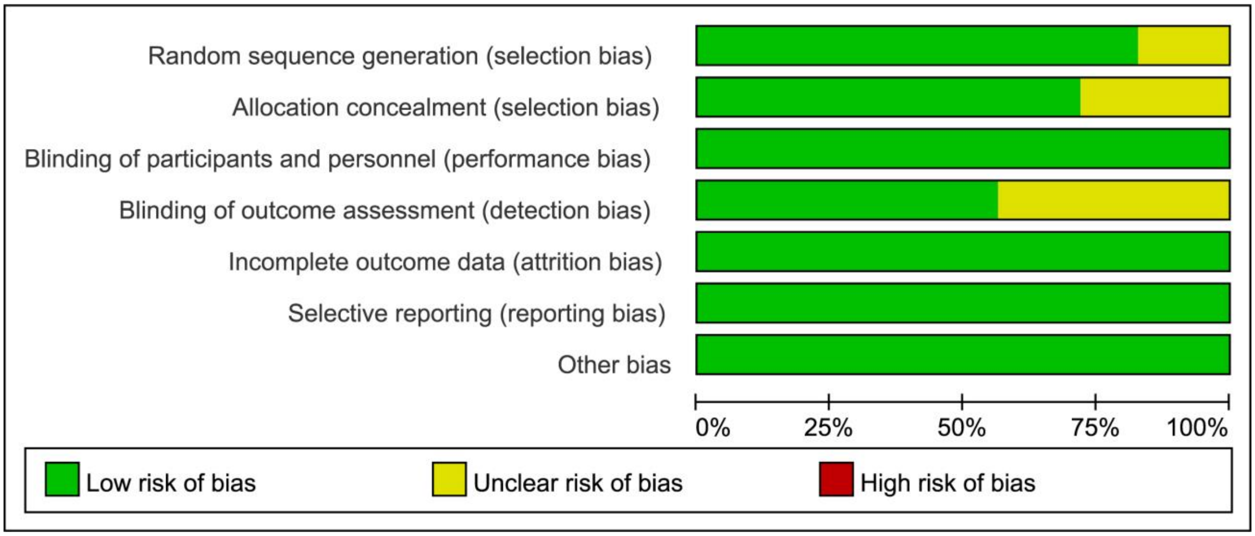

Supplementary Figure 1 *Risk of bias assessment. Overall risk of bias, using Cochrane's risk of bias assessment tool.*

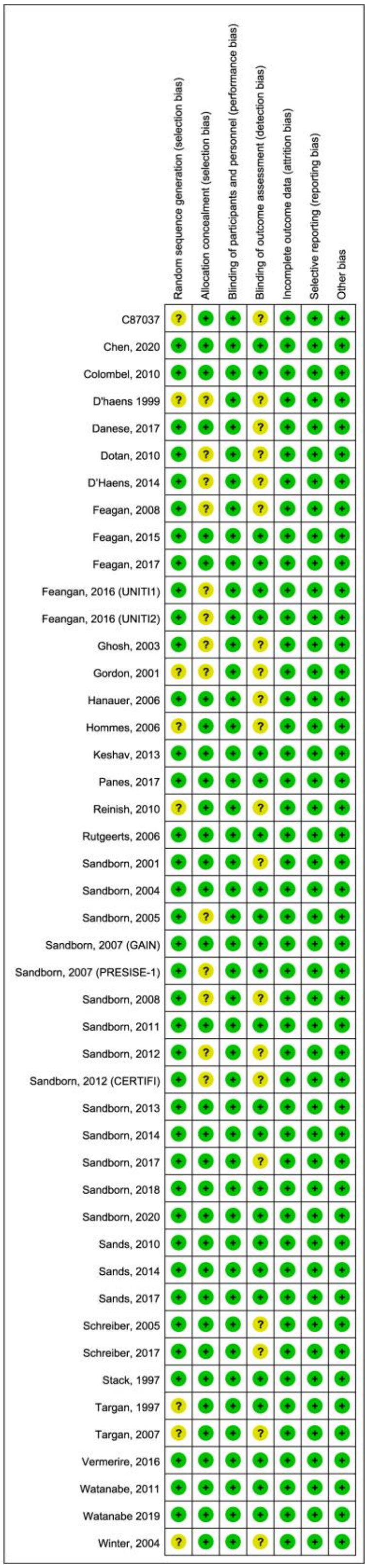

Supplementary Figure 2 *Risk of bias assessment. Study-level risk of bias, using Cochrane's risk of bias assessment tool.*

Model Development

After graphics exploration, the longitudinal profiles of efficacy outcomes were characterized using a hierarchical regression model with maximum likelihood estimation method. A nonparametric method was implemented to model the outcomes from placebo arms to obtain estimation of placebo effects at each trial and at each time point, so as to avoid misestimation of placebo effects and reduce biases due to placebo model misspecification. The general model could be described as:

$$E_{ijt} = E_{0it} + E_{\text{drug}} \quad (1)$$

$$E_{\text{drug}} = f(\text{drug}, \text{dose}, \text{regimen}, \text{time}, \theta, X_{ij}) \quad (2)$$

Where E_{ijt} represents the efficacy outcome in *j*th treatment arm of *i*th trial at *i*time, which is the sum of E_{0it}(the placebo effects of *i*th trial at *t*time), and E_{drug}(the drug effects in *j*th treatment arm of *i*th trial at *t*time). Drug effects (E_{drug}) is a function dependent on type of drug, dose, regimen, time, fixed-effect model parameters θ, and covariates *X*.

At first, the drug effects were set not to change with time. Then, in the process of modeling, if the fit degree of the model was improved, a time variable was added to create a nonlinear model to describe the time-varying drug effects, showing as follows:

$$E_{\text{drug}} = E_{\text{max}_{\text{drug}}} \cdot (1 - e^{-k \cdot \text{time}}) \quad (3)$$

Where $E_{max_{drug}}$ presented the maximum efficacy of each treatment. The parameter *k*, as the onset rate constant of drug effect, was initially estimated as a shared parameter for all treatments. With the assumption that drugs in the same class shares similar active mechanism, with a common saturation relationship. Drugs sharing a same pathway were also thought to have a similar absorption process. For drugs without enough data for estimation of a common rate constant, a shared parameter for a drug class or a route was used in the time-varying model. Both shared and separated parameters were acceptable in our model development, only the best fitted one was used for the time-varying drug effect in the final model.

During the model development, the maximum efficacy of each treatment was initially incorporated to be constant over dose and described by a scaling factor, E_{max}. Then, the parameter E_{max} was separated into several parameters matching different doses, routes, and

regimens of a drug. For drugs with a dose range, a dose-response relationship was estimated with linear, Emax, and sigmoid Emax model. Functions of these models are listed as follows:

1 linear model

$$f = E_0 + \delta \cdot \text{dose} \tag{4}$$

2 Emax model

$$f = E_0 + \frac{E_{\text{max}} \cdot \text{dose}}{\text{ED}_{50} + \text{dose}} \tag{5}$$

3 sigmoid Emax model

$$f = E_0 + \frac{E_{\text{max}} \cdot \text{dose}^h}{\text{ED}_{50}{}^h + \text{dose}^h} \tag{6}$$

Where E_0 represented the placebo efficacy, while E_{max}represented the maximum efficacy of the drug. δ was the slope parameter of linear model. *h* was the slope parameter of Emax model, called Hill factor.

Model for ΔCDAI, ΔCRP, ΔIBDQ

The model for ΔCDAI, ΔCRP, and ΔIBDQ was very similar, which can be shown as:

$$\Delta Y_{ijt} = E_{0it} + E_{\text{drug}ijt} \tag{7}$$

where ΔY_{ijt} represented the values of ΔCDAI, ΔCRP, and ΔIBDQ at *t* time in *j* treatment arm of *i* trial. E_{0ij} represented the placebo effect of *i* trial at *t* time. $E_{\text{drug}ijt}$ represented the drug effects of *j* treatment arm in *i* trial at *t* time.

Weight was calculated based on the inverse of the variance for each arm, and the sample size (*N*) of the treatment arm was also taken into consideration, as shown in the following equation:

$$\text{Weight} = \frac{SD}{\sqrt{N}} \tag{8}$$

SD and *N* were considered when calculating weights to ensure that more accurate and larger sample size outcomes had a greater impact on the final model.

Model for CDAI-100, CDAI-70, CDAI150

$$N_{\text{effect}ijt} \sim \text{binomial}\,(P_{ijt}, N_{ij}) \tag{9}$$

$$P_{ijt} = g(E_{0it} + E_{\text{drug}ijt}) \tag{10}$$

For ratio outcomes, efficacy of a treatment arm was considered as P_{ijt} (the probability of patients achieving endpoints at *t* time in *j* treatment arm of *i* trial), from a binomial distribution of $N_{\text{effect}ijt}$(the number of patients achieving endpoint at *t* time in *j* treatment arm of *i* trial) with probability (P_{ijt}) and sample size (N_{ij}). *g* was a logit translate of the efficacy of treatment, which was a sum of E_{0it}(placebo effect of *i* trial at *t* time) and $E_{\text{drug}ijt}$(drug effect of *j* treatment arm in *i* trial at *t* time), to limit the probability to a range of 0-1.

For CDAI-100, CDAI-70, CDAI150, the weight was based on the standard error of observed values and was generated by the following equation with *P* and *N*, avoiding the possible deviations in the final model cause by the extreme outcome values, and ensuring that more larger sample size outcomes had a greater impact.

$$\text{Weight} = \sqrt{\frac{P \cdot (1 - P)}{N}} \tag{11}$$

Covariate

Baseline characteristics, including age, percentage of male, duration of Crohn's Disease, smoking status, CDAI, IBDQ, CRP, were considered as the covariate in the model. In the process of model development, covariates were investigated for their possible impact on the treatment efficacies by the following function where θ was introduced as the parameter quantifying the impact of baseline characteristics on the treatment efficacies.

$$\text{Effect}_{\text{Covariate}} = \frac{\text{Covariate}^{\theta}}{\text{mean (Covariate)}} \tag{12}$$

Final Models

Parameters and model fitted time-course plots CDAI150 model

Supplementary Table 2 Parameter estimates for CDAI150 model.

Parameter	Route (regimen)	Estimate	95%CI
**E_{max}**			
Infliximab	i.v. (5 mg/kg)<br>i.v. (5 mg/kg 0, 2, 6, q8w)	1.16	(0.87, 1.46)
Natalizumab	i.v. (3mg/kg)<br>i.v. (3, 6 mg/kg q4w)<br>i.v. (300mg q4w)	0.44	(0.31, 0.57)
CDP571	i.v. (10 mg/kg q8w)	0.18	(-0.14, 0.50)
Etanercept	s.c. (25 mg biw)	-0.70	(-1.68, 0.27)
Certolizumab pegol	s.c. (100, 200, 400 mg q4w)<br>s.c. (200, 400 mg q2w)<br>s.c. (400 mg 0, 2, 4, q4w)	0.47	(0.31, 0.63)
Adalimumab (slope)[a]	s.c. (40, 80, 160 mg 0w followed by 20, 40, 80 mg 2w)<br>s.c. (160 mg 0w followed by 80 mg 2w followed by 40 mg 4, 6w)	6.00×10^{-3}	(4.22×10^{-3}, 7.78×10^{-3})
Onercept	s.c. (10, 25, 35, 50 mg tiw)	0.18	(-0.56, 0.92)
Vedolizumab	i.v. (0.5, 2 mg/kg q4w)<br>i.v. (300 mg 0, 2, 6w)	0.70	(0.45, 0.95)
Ustekinumab	s.c. (90 mg qw)	0.44	(-0.45, 1.33)
Apilimod	p.o. (50, 100 mg qd)	-0.12	(-0.55, 0.31)
Andecaliximab	s.c. (150 mg q2w)<br>s.c. (150, 300 mg qw)	-0.26	(-1.40, 0.87)
Tofacitinib	p.o. (1, 5, 10, 15 mg bid)	0.76	(-1.14, 2.66)
Filgotinib	p.o. (200 mg qd)	1.50	(-1.02, 4.01)
Risankizumab (slope)[a]	i.v. (200, 600 mg q4w)	2.55×10^{-3}	(8.88×10^{-4}, 4.22×10^{-3})
PF-04236921 (slope)[a]	s.c. (10, 50, 200 mg q4w)	8.33×10^{-3}	(2.92×10^{-3}, 1.37×10^{-2})
Brazikumab	i.v. (700 mg q4w)	0.69	(-0.04, 1.42)
Upadacitinib	p.o. (3, 6, 12, 24 mg bid)<br>p.o. (24 mg qd)	1.09	(-0.39, 2.58)
Fontolizumab	i.v.-s.c. (1, 4 mg/kg 0w followed by 0.1, 1 mg/kg q4w)<br>i.v. (4, 10 mg/kg q4w)	0.78	(0.39, 1.16)
Abrilumab	s.c. (21, 70, 210 mg 0, 1, 2, q4w)	0.51	(-0.03, 1.06)
Vercirnon	p.o. (250, 500 mg qd)<br>p.o. (250, 500 mg bid)	-0.19	(-0.48, 0.11)
Laquinimod	p.o. (0.5, 1, 1.5, 2 mg qd)	0.88	(0.45, 1.32)
Ontamalimab	s.c. (22.5, 75, 225 mg q4w)	0.45	(-0.13, 1.02)
**k_{JAK}**			
Rate constant for the onset of JAK inhibitor	NA	0.11	(0.01, 1.40)
**Covariate**			
Baseline CDAI	NA	-5.22	(-7.90, -2.54)
Baseline CRP	NA	0.51	(0.20, 0.83)

CDAI150, an absolute CDAI score of less than 150; CDAI, Crohn's Disease Activity Index; 95%CI, 95% confidence interval; Emax, maximum drug efficacy; NA, not available; qd, once daily; bid, twice daily; qw, once weekly; tiw, 3 times weekly; q2w, once every 2 weeks; q4w, once every 4 weeks; q8w, once every 8 weeks.

[a] *The dose-response relationship is linear, which means that the Emax of any dose of the drug = drug (slope) * dose.*

[b] *The dose-response relationship is* E_{max} *model, which means that the Emax of any dose of the drug = drug (Emax) * dose / (drug (ED50) * dose).*

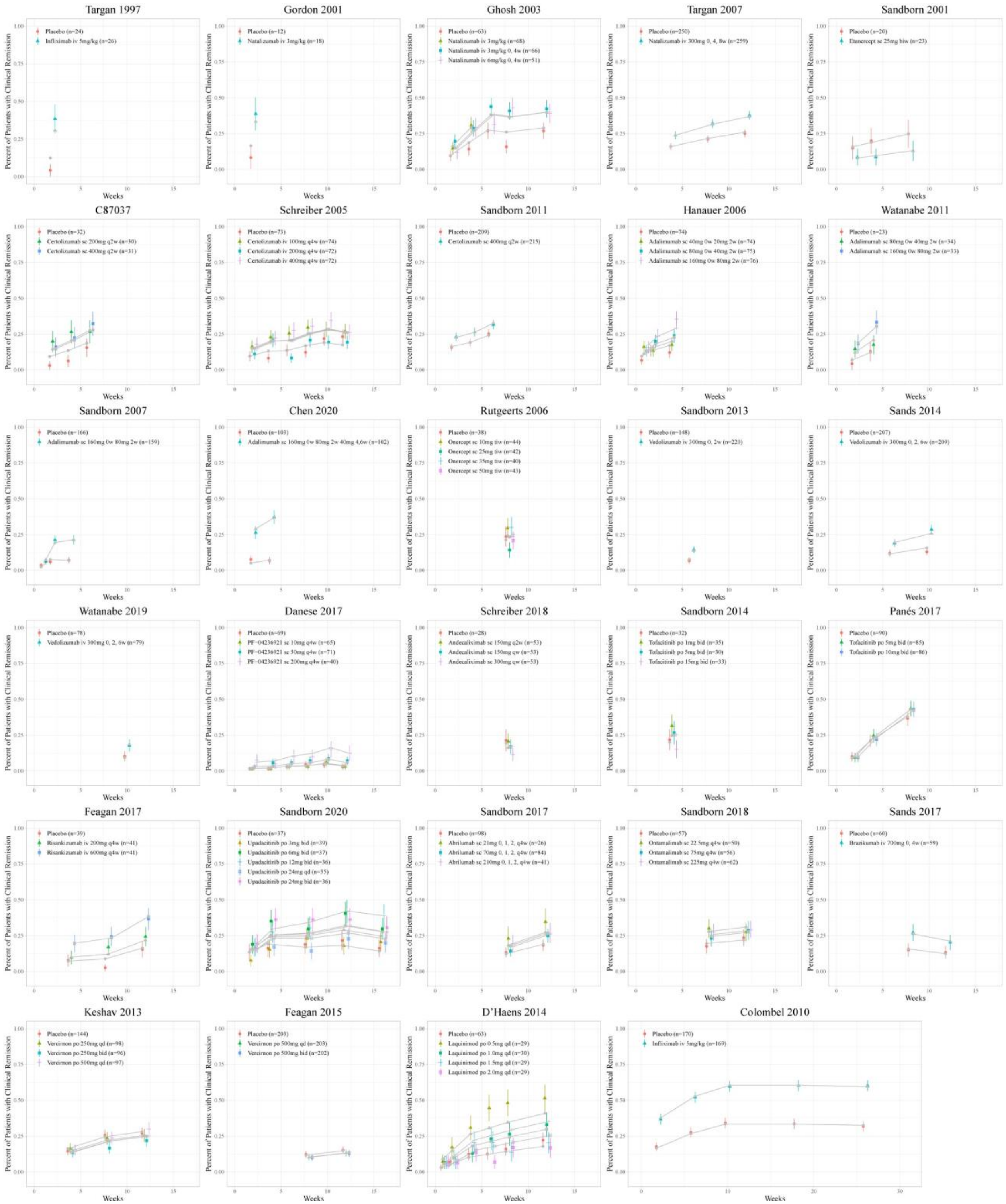
Targan 1997
Gordon 2001
Ghosh 2003
Targan 2007
Sandborn 2001
C87037
Schreiber 2005
Sandborn 2011
Hanauer 2006
Watanabe 2011
Sandborn 2007
Chen 2020
Rutgeerts 2006
Sandborn 2013
Sands 2014
Watanabe 2019
Danese 2017
Schreiber 2018
Sandborn 2014
Panés 2017
Feagan 2017
Sandborn 2020
Sandborn 2017
Sandborn 2018
Sands 2017
Keshav 2013
Feagan 2015
D'Haens 2014
Colombel 2010
Percent of Patients with Clinical Remission
Weeks

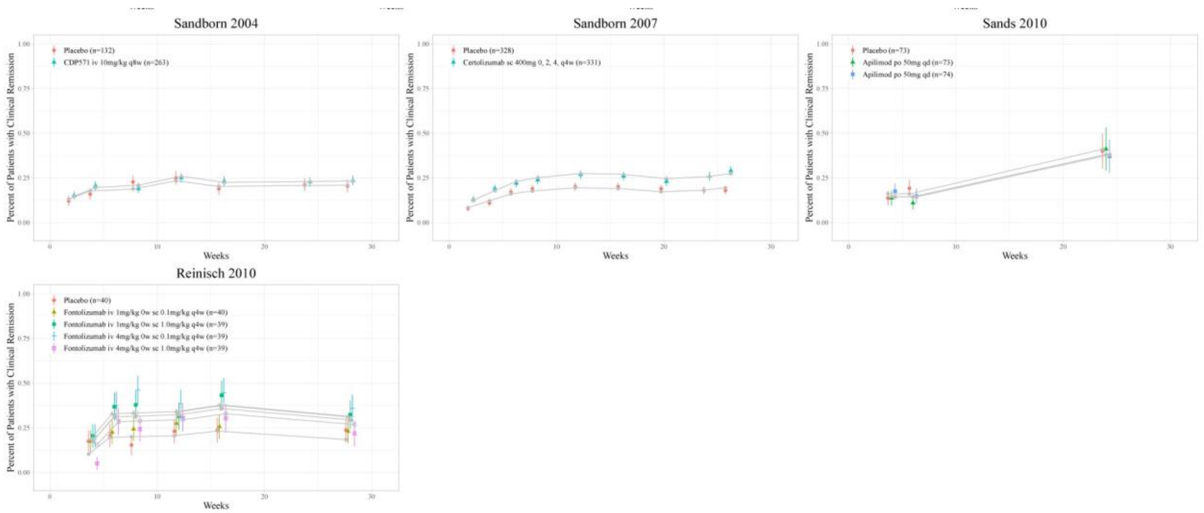

***Supplementary Figure 3** Model fitted time-course plots of response rate for CDAI150. Color symbols and vertical bars are observed mean and calculated weight of time points; gray symbols and lines are the model predictions. CDAI150, an absolute CDAI score of less than 150; CDAI, Crohn's Disease Activity Index; qd, once daily; bid, twice daily; qw, once weekly; tiw, three times weekly; q2w, once every 2 weeks; q4w, once every 4 weeks.*

Parameters and model fitted time-course plots CDAI-100 model

Supplementary Table 3 Parameter estimates for CDAI-100 model.

Parameter	Route (regimen)	Estimate	95%CI
**E_{max}**			
Infliximab	i.v. (5 mg/kg 0, 2, 6, q8w)	1.05	(0.83, 1.28)
Natalizumab	i.v. (300mg q4w)	0.57	(0.34, 0.80)
Certolizumab pegol	s.c. (200, 400 mg q2w)<br>s.c. (400 mg 0, 2, 4, q4w)	0.43	(0.22, 0.64)
Adalimumab (Emax)[a]	s.c. (40, 80, 160 mg 0w followed by 20, 40, 80 mg 2w)	0.93	(0.30, 1.56)
Adalimumab (ED50)[a]	NA	34.13	(1.66, 703.51)
Onercept	s.c. (10, 25, 35, 50 mg tiw)	0.04	(-0.61, 0.69)
Vedolizumab	i.v. (0.5, 2 mg/kg q4w)<br>i.v. (300 mg 0, 2, 6w)	0.62	(0.39, 0.85)
Ustekinumab	s.c. (90 mg qw)	0.51	(-0.16, 1.18)
Apilimod	p.o. (50, 100 mg qd)	-0.35	(-0.76, 0.06)
Tofacitinib	p.o. (1, 5, 10, 15 mg bid)	0.61	(0.15, 1.08)
Filgotinib	p.o. (200 mg qd)	0.44	(0.02, 0.85)
PF-04236921	s.c. (10, 50, 200 mg q4w)	0.53	(0.12, 0.94)
Brazikumab	i.v. (700 mg q4w)	0.95	(0.19, 1.71)
Upadacitinib (slope)[b]	p.o. (3, 6, 12, 24 mg bid)<br>p.o. (24 mg qd)	4.34×10^{-2}	(6.32×10^{-3}, 8.04×10^{-2})
Fontolizumab	i.v.-s.c. (1, 4 mg/kg 0w followed by 0.1, 1 mg/kg q4w)<br>i.v. (4, 10 mg/kg q4w)	0.40	(0.06, 0.75)
Vercirnon	p.o. (250, 500 mg qd)<br>p.o. (250, 500 mg bid)	0.15	(-0.08, 0.37)
Laquinimod	p.o. (0.5, 1, 1.5, 2 mg qd)	0.51	(0.10, 0.93)
Ontamalimab	s.c. (22.5, 75, 225 mg q4w)	0.23	(-0.18, 0.64)
**Covariate**			
Baseline CDAI	NA	-3.21	(-7.39, 0.96)
Baseline CRP	NA	0.29	(-0.14, 0.71)

CDAI-100, a reduction of at least 100 points in the CDAI score; CDAI, Crohn's Disease Activity Index; 95%CI, 95% confidence interval; Emax, maximum drug efficacy; NA, not available; qd, once daily; bid, twice daily; qw, once weekly; tiw, 3 times weekly; q2w, once every 2 weeks; q4w, once every 4 weeks; q8w, once every 8 weeks.

[a] *The dose-response relationship is E_{max} model, which means that the Emax of any dose of the drug = drug (Emax) * dose / (drug (ED50) * dose).*

[b] *The dose-response relationship is linear, which means that the Emax of any dose of the drug = drug (slope) * dose.*

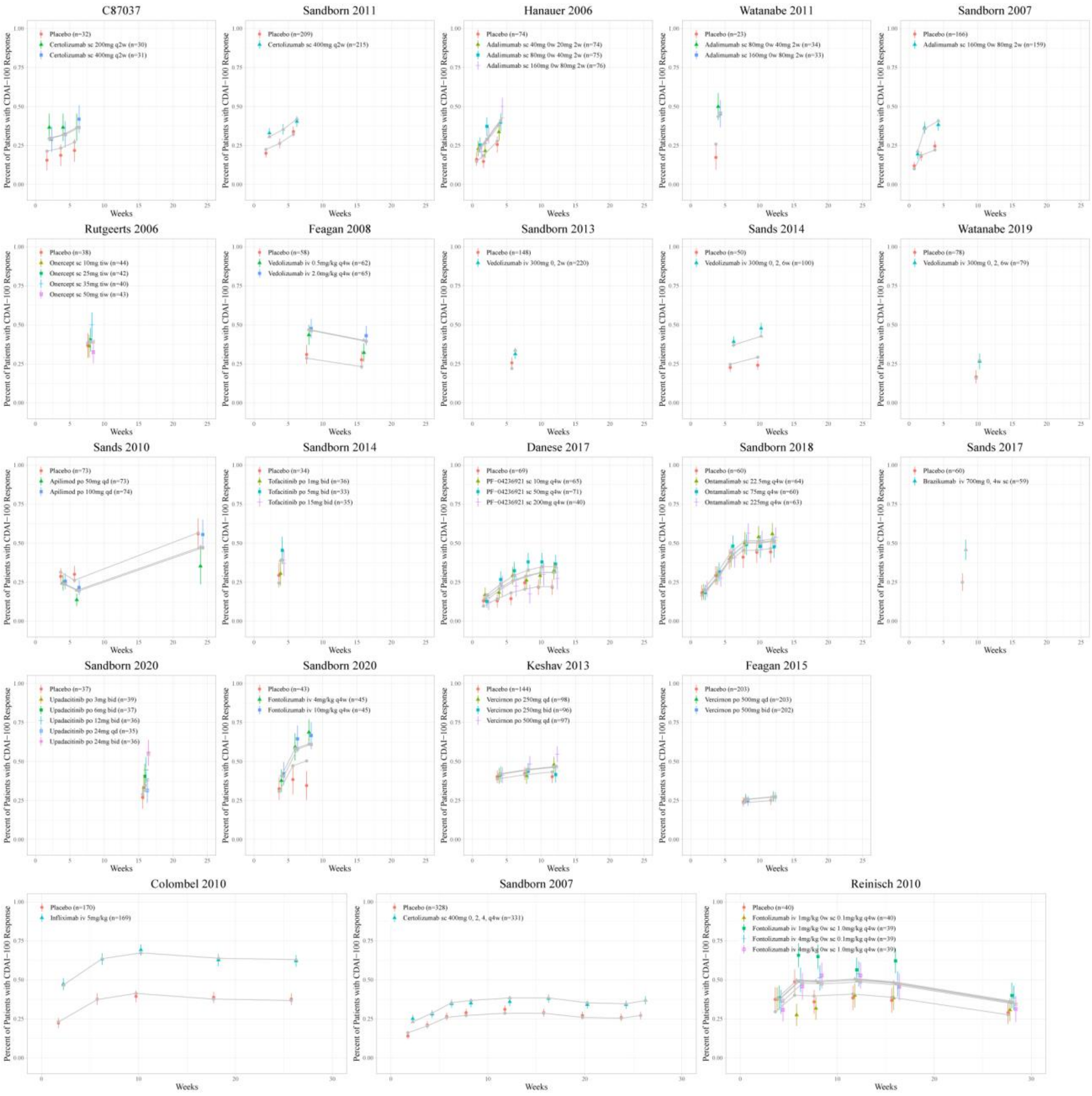

Supplementary Figure 4 *Model fitted time-course plots of response rate for CDAI-100. Color symbols and vertical bars are observed mean and calculated weight of time points; gray symbols and lines are the model predictions. CDAI-100, reduction of at least 100 points in the CDAI score; CDAI, Crohn's Disease Activity Index; qd, once daily; bid, twice daily; tiw, three times weekly; q2w, once every other week; q4w, once every 4 weeks.*

Parameters and model fitted time-course plots CDAI-70 model

Supplementary Table 4 Parameter estimates for CDAI-70 model.

Parameter	Route (regimen)	Estimate	95%CI
**E_{max}**			
Infliximab	i.v. (5 mg/kg)<br>i.v. (5 mg/kg 0, 2, 6, q8w)	1.20	(0.96, 1.44)
Natalizumab	i.v. (3mg/kg)<br>i.v. (3, 6 mg/kg q4w)	0.48	(0.33, 0.63)
CDP571	i.v. (10 mg/kg q8w)	0.41	(0.09, 0.72)
Etanercept	s.c. (25 mg biw)	0.31	(-0.58, 1.21)
Certolizumab pegol	s.c. (200, 400 mg q2w)<br>s.c. (400 mg 0, 2, 4, q4w)	0.49	(0.26, 0.71)
Adalimumab	s.c. (40, 80, 160 mg 0w followed by 20, 40, 80 mg 2w)<br>s.c. (160 mg 0w followed by 80 mg 2w followed by 40 mg 4, 6w)	0.87	(0.62, 1.11)
Vedolizumab	i.v. (0.5, 2 mg/kg q4w)	0.34	(-0.06, 0.73)
Ustekinumab	s.c. (90 mg qw)	0.23	(-0.62, 1.08)
Semapimod	i.v. (60 mg for 3d)	0.04	(-0.51, 0.59)
Tofacitinib	p.o. (1, 5, 10, 15 mg bid)	0.32	(-0.11, 0.75)
PF-04236921	s.c. (10, 50, 200 mg q4w)	0.71	(0.28, 1.15)
Brazikumab	i.v. (700 mg q4w)	0.23	(-0.48, 0.95)
Upadacitinib (Emax)[a]	p.o. (3, 6, 12, 24 mg bid)<br>p.o. (24 mg qd)	0.89	(0.09, 1.69)
Upadacitinib (ED50)[a]	NA	3.91	(0.19, 79.30)
Vercirnon	p.o. (250, 500 mg qd)<br>p.o. (250 mg bid)	0.26	(-0.06, 0.58)
Laquinimod	p.o. (0.5, 1, 1.5, 2 mg qd)	0.37	(-0.07, 0.80)
Ontamalimab	s.c. (22.5, 75, 225 mg q4w)	0.11	(-0.32, 0.54)
**Covariate**			
Baseline CDAI	NA	-2.03	(-5.37, 1.32)
Baseline CRP	NA	0.27	(-0.12, 0.67)

CDAI-70, reduction of at least 70 points in the CDAI score; CDAI, Crohn's Disease Activity Index; 95%CI, 95% confidence interval; Emax, maximum drug efficacy; NA, not available; qd, once daily; bid, twice daily; q4w, once every 4 weeks; q8w, once every 8 weeks.

*[a] The dose-response relationship is E_{max} model, which means that the Emax of any dose of the drug = drug (Emax) * dose / (drug (ED50) * dose).*

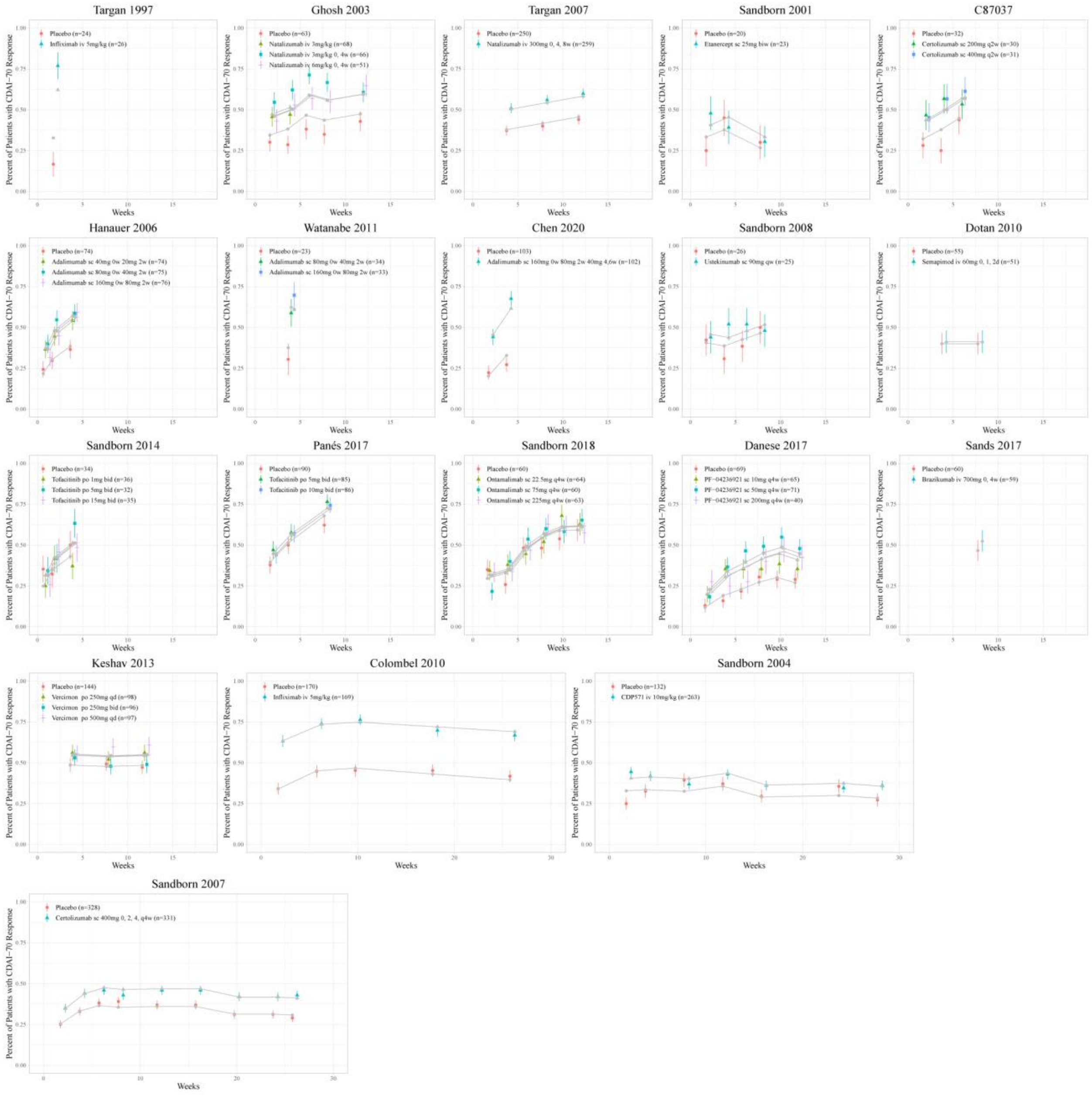

Supplementary Figure 5 *Model fitted time-course plots of response rate for CDAI-70. Color symbols and vertical bars are observed mean and calculated weight of time points; gray symbols and lines are the model predictions. CDAI-70, reduction of at least 70 points in the CDAI score; CDAI, Crohn's Disease Activity Index; qd, once daily; bid, twice daily; q2w, once every other week; q4w, once every 4 weeks.*

Parameters and model fitted time-course plots ΔCDAI model

Supplementary Table 5 Parameter estimates for ΔCDAI model.

Parameter	Route (regimen)	Estimate	95%CI
**E_{max}**			
Natalizumab	i.v. (300 mg q4w)	-51.36	(-70.89, -31.83)
CDP571	i.v. (10 mg/kg q8w)	-18.26	(-33.68, -2.85)
Etanercept	s.c. (25 mg/kg biw)	-15.93	(-84.16, 52.30)
Certolizumab pegol	s.c. (400 mg q2w)<br>s.c. (400 mg 0, 2, 4, q4w)	-31.53	(-45.48, -17.59)
Adalimumab (Emax)[a]	s.c. (40, 80, 160 mg 0w followed by 20, 40, 80 mg 2w)<br>s.c. (160 mg 0w followed by 80 mg 2w followed by 40 mg 4, 6w)	-151.24	(-322.11, 19.64)
Adalimumab (ED50)[a]	NA	112.23	(9.02, 1.40×10^3)
Vedolizumab	i.v. (0.5, 2 mg/kg q4w)	-22.38	(-49.72, 4.97)
Tofacitinib	p.o. (1, 5, 10, 15 mg bid)	-26.23	(-49.46, -2.99)
Filgotinib	p.o. (200 mg qd)	-32.16	(-69.00, 4.68)
Abatacept	i.v. (3, 10 mg/kg 0, 2, 4, 10w)<br>i.v. (30 mg/kg 0, 2w followed by 10 mg/kg 4, 10w)	-20.19	(-43.15, 2.76)
Risankizumab	i.v. (200, 600 mg q4w)	-81.85	(-122.85, -40.86)
PF-04236921	s.c. (10, 50, 200 mg q4w)	-31.11	(-60.23, -1.99)
Brazikumab	i.v.-s.c. (700 mg 0, 4w)	-39.99	(-85.67, 5.70)
Fontolizumab	i.v. (4, 10 mg/kg q4w)<br>i.v.-s.c. (1, 4 mg/kg 0w followed by 0.1, 1 mg/kg q4w)	-41.00	(-66.47, -15.52)
Abrilumab	s.c. (21, 70, 210 mg 0, 2, 4, q4w)	-35.63	(-41.90, -29.36)
Vercirnon	p.o. (250, 500 mg qd)<br>p.o. (250 mg bid)	-17.62	(-50.29, 15.06)
**$k_{general}$**			
Rate constant for the onset of all drugs	NA	0.22	(0.14, 0.34)
**Covariate**			
Baseline CDAI	NA	-2.05	(-4.93, 0.83)

ΔCDAI, change form baseline in CDAI; CDAI, Crohn's Disease Activity Index; 95%CI, 95% confidence interval; Emax, maximum drug efficacy*; kgeneral, rate constant for the onset of all drugs except for infliximab; NA, not available; qd, once daily; bid, twice daily; biw, twice weekly; q2w, once every 2 weeks; q4w, once every 4 weeks.*

[a] *The dose-response relationship is E_{max} model, which means that the Emax of any dose of the drug = drug (Emax) * dose / (drug (ED50) * dose).*

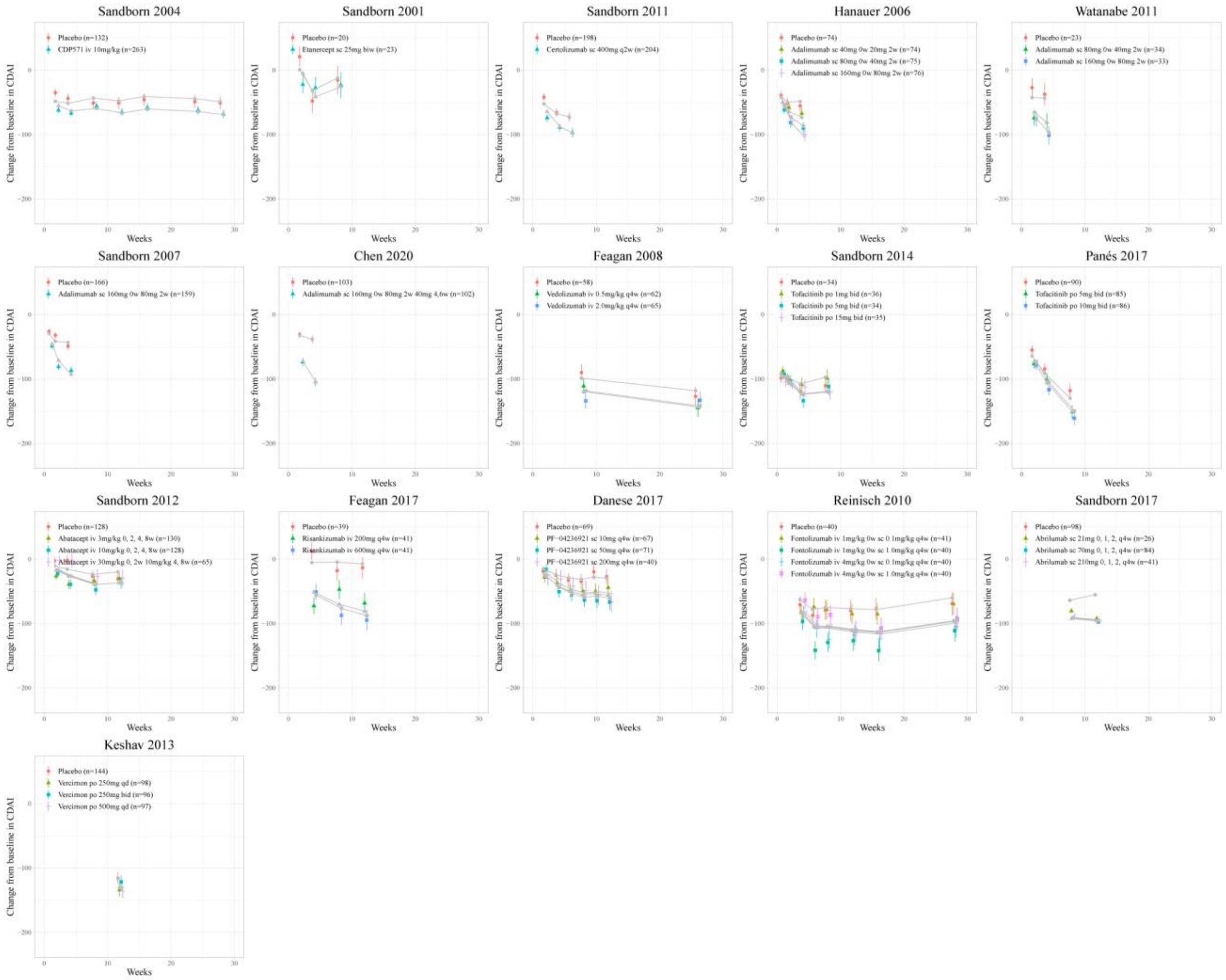

Supplementary Figure 6 *Model fitted time-course plots of CDAI change from baseline. Color symbols and vertical bars are observed mean and calculated weight of time points; gray symbols and lines are the model predictions. CDAI, Crohn's Disease Activity Index; qd, once every day; bid, twice every day; biw, twice weekly; q4w, once every 4 weeks; q6w, once every 6 weeks.*

Parameters and model fitted time-course plots ΔCRP model

Supplementary Table **6 Parameter estimates for ΔCRP model.**

Parameter	Route (regimen)	Estimate	95%CI
**E_{max}**			
Infliximab	i.v. (5 mg/kg)	-0.61	(-0.98, -0.24)
Natalizumab	i.v. (300 mg q4w)	-0.89	(-1.12, -0.65)
CDP571	i.v. (10 mg/kg)	-0.15	(-0.28, -0.02)
Certolizumab pegol (slope)[b]	s.c. (400 mg q2w)<br>s.c. (100, 200, 400 mg q4w)<br>s.c. (400 mg 0, 2, 4, q4w)	1.12×10^{-2}	(-1.70×10^{-2}, -5.47×10^{-4})
Adalimumab	s.c. (40, 80, 160 mg 0w followed by 20, 40, 80 mg 2w)<br>s.c. (160 mg 0w followed by 80 mg 2w followed by 40 mg 4, 6w)	-0.47	(-0.67, -0.27)
Vedolizumab	i.v. (300 mg 0, 2, 6w)	0.03	(-0.45, 0.52)
Ustekinumab	s.c. (90 mg)	-0.22	(-0.54, 0.10)
Semapimod	i.v. (60 mg for 3d)	-0.07	(-1.80, 1.66)
Tofacitinib	p.o. (1, 5, 10, 15 mg bid)	-0.07	(-0.10, -0.04)
Abatacept	i.v. (3, 10 mg/kg 0, 2, 4, 10w)<br>i.v. (30 mg/kg 0, 2w followed by 10 mg/kg 4, 10w)	0.59	(0.32, 0.87)
PF-04236921 (Emax)[a]	s.c. (10, 50, 200 mg q4w)	-7.47	(-12.76, -2.18)
PF-04236921 (ED50)[a]	NA	93.69	(41.26, 241.86)
Upadacitinib (slope)[b]	p.o. (3, 6, 12, 24 mg bid)<br>p.o. (24 mg qd)	-0.22	(-0.43, -0.01)
Fontolizumab	i.v. (4, 10 mg/kg q4w)<br>i.v.-s.c. (1, 4 mg/kg 0w followed by 0.1, 1 mg/kg q4w)	-0.13	(-0.28, 0.03)
Vercirnon	p.o. (250, 500 mg qd)<br>p.o. (250, 500 mg bid)	0.10	(-0.05, 0.25)
Laquinimod	p.o. (0.5, 1, 1.5, 2 mg qd)	0.25	(-0.01, 0.50)
Risankizumab	i.v. (200, 600 mg q4w)	4.60×10^{-3}	(5.70×10^{-2}, 4.78×10^{-2})
Ontamalimab	s.c. (22.5, 75, 225 mg q4w)	-0.05	(-0.12, 0.02)
**Covariate**			
Age	NA	-7.69	(-11.11, -4.26)
Disease duration	NA	4.95	(3.60, 6.29)
Baseline CRP	NA	-0.87	(-1.24, -0.50)

ΔCRP, change form baseline in CRP; CRP, C-reactive protein; 95%CI, 95% confidence interval; Emax, maximum drug efficacy; NA, not available; qd, once daily; bid, twice daily; q2w, once every 2 weeks; q4w, once every 4 weeks.

[a] *The dose-response relationship is E_{max} model, which means that the Emax of any dose of the drug = drug (Emax) * dose / (drug (ED50) * dose).*

[b] *The dose-response relationship is linear, which means that the Emax of any dose of the drug = drug (slope) * dose.*

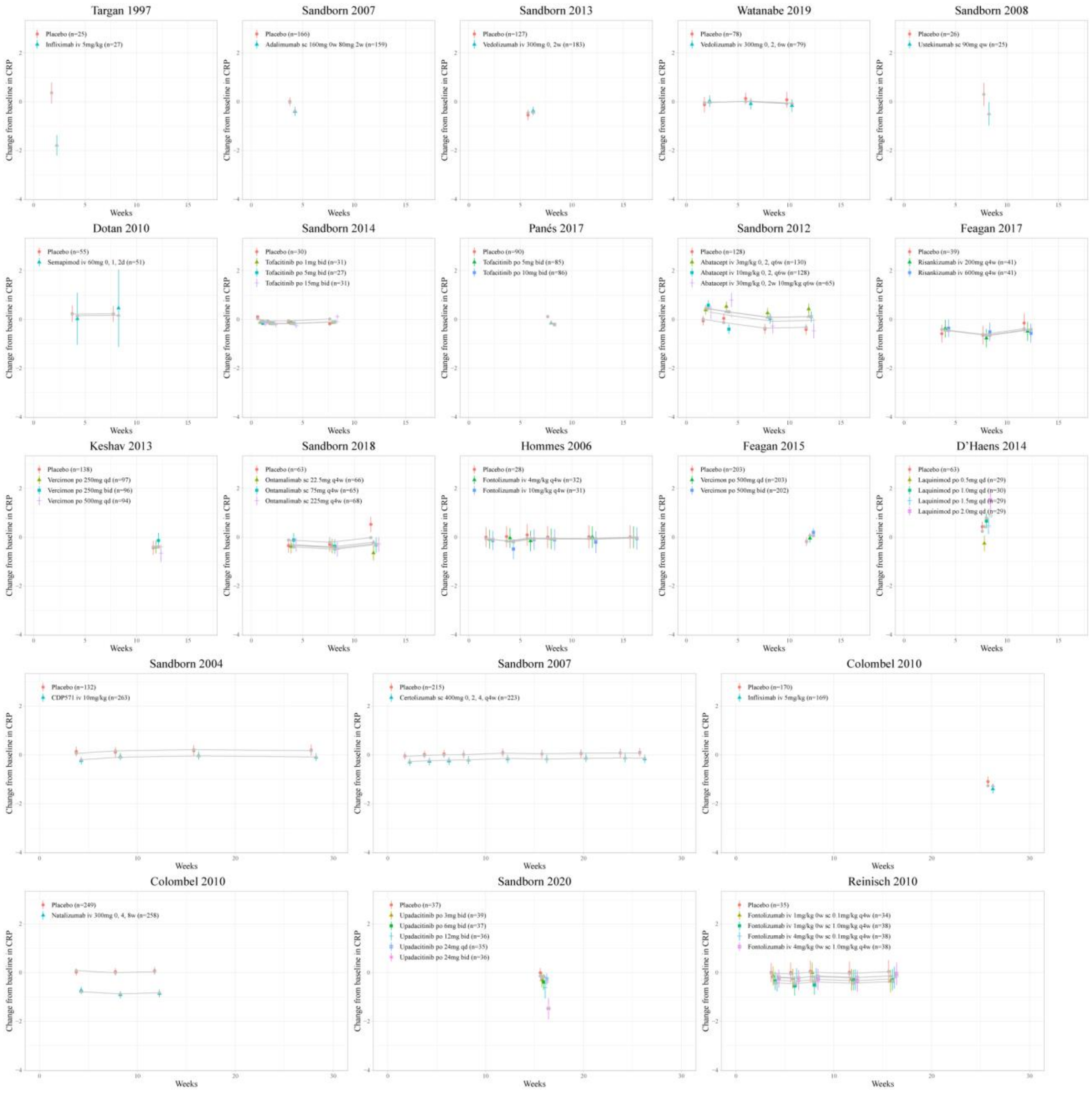

Supplementary Figure 7 *Model fitted time-course plots of CRP change from baseline. Color symbols and vertical bars are observed mean and calculated weight of time points; gray symbols and lines are the model predictions. CRP, C-reactive protein; qd, once daily; bid, twice daily; q2w, once every 2 weeks; q4w, once every 4 weeks.*

Parameters and model fitted time-course plots ΔIBDQ model

Supplementary Table 7 Parameter estimates for ΔIBDQ model.

Parameter	Route (regimen)	Estimate	95%CI
**E_{max}**			
Infliximab	i.v. (5, 10, 20 mg/kg)<br>i.v. (5 mg/kg 0, 2, 6, q8w)	15.72	(7.59, 23.85)
Natalizumab	i.v. (3mg/kg)<br>i.v. (3, 6 mg/kg q4w)	2.72	(0.66, 4.79)
CDP571	i.v. (10 mg/kg q8w)	3.93	(0.55, 7.31)
Etanercept	s.c. (25 mg biw)	-1.51	(-26.40, 23.38)
Certolizumab pegol	s.c. (400 mg 0, 2, 4, q4w)	0.31	(-0.19, 0.82)
Adalimumab (slope)[a]	s.c. (40, 80, 160 mg 0w followed by 20, 40, 80 mg 2w)	0.10	(0.05, 0.15)
Vedolizumab	i.v. (0.5, 2 mg/kg q4w)	0.97	(-1.80, 3.75)
Semapimod	i.v. (60 mg for 1, 3d)	-1.30	(-27.19, 24.60)
Apilimod	p.o. (50, 100 mg qd)	-8.26	(-41.19, 24.68)
Filgotinib	p.o. (200mg qd)	6.74	(-0.05, 13.54)
Tofacitinib	p.o. (1, 5, 10, 15 mg bid)	52.74	(39.23, 66.25)
Abatacept	i.v. (3, 10 mg/kg 0, 2, 4, 10w)<br>i.v. (30 mg/kg 0, 2w followed by 10 mg/kg 4, 10w)	-3.54	(-4.35, -2.72)
Risankizumab (slope)[a]	i.v. (200, 600 mg q4w)	0.05	(0.01, 0.10)
Upadacitinib	p.o. (3, 6, 12, 24 mg bid)<br>p.o. (24 mg qd)	4.70	(-1.57, 10.97)
Fontolizumab	i.v.-s.c. (1, 4 mg/kg 0w followed by 0.1, 1 mg/kg q4w)	-0.34	(-4.02, 3.35)
Vercirnon	p.o. (500 mg qd)<br>p.o. (500 mg bid)	3.54	(-5.84, 12.93)
**Covariate**			
Disease duration	NA	-8.98	(-10.60, -7.36)

ΔIBDQ, change form baseline in IBDQ; IBDQ, inflammatory bowel disease questionnaire; 95%CI, 95% confidence interval; Emax, maximum drug efficacy; NA, not available; qd, once daily; bid, twice daily; q4w, once every 4 weeks; q8w, once every 8 weeks.

[a] *The dose-response relationship is linear, which means that the Emax of any dose of the drug = drug (slope) * dose.*

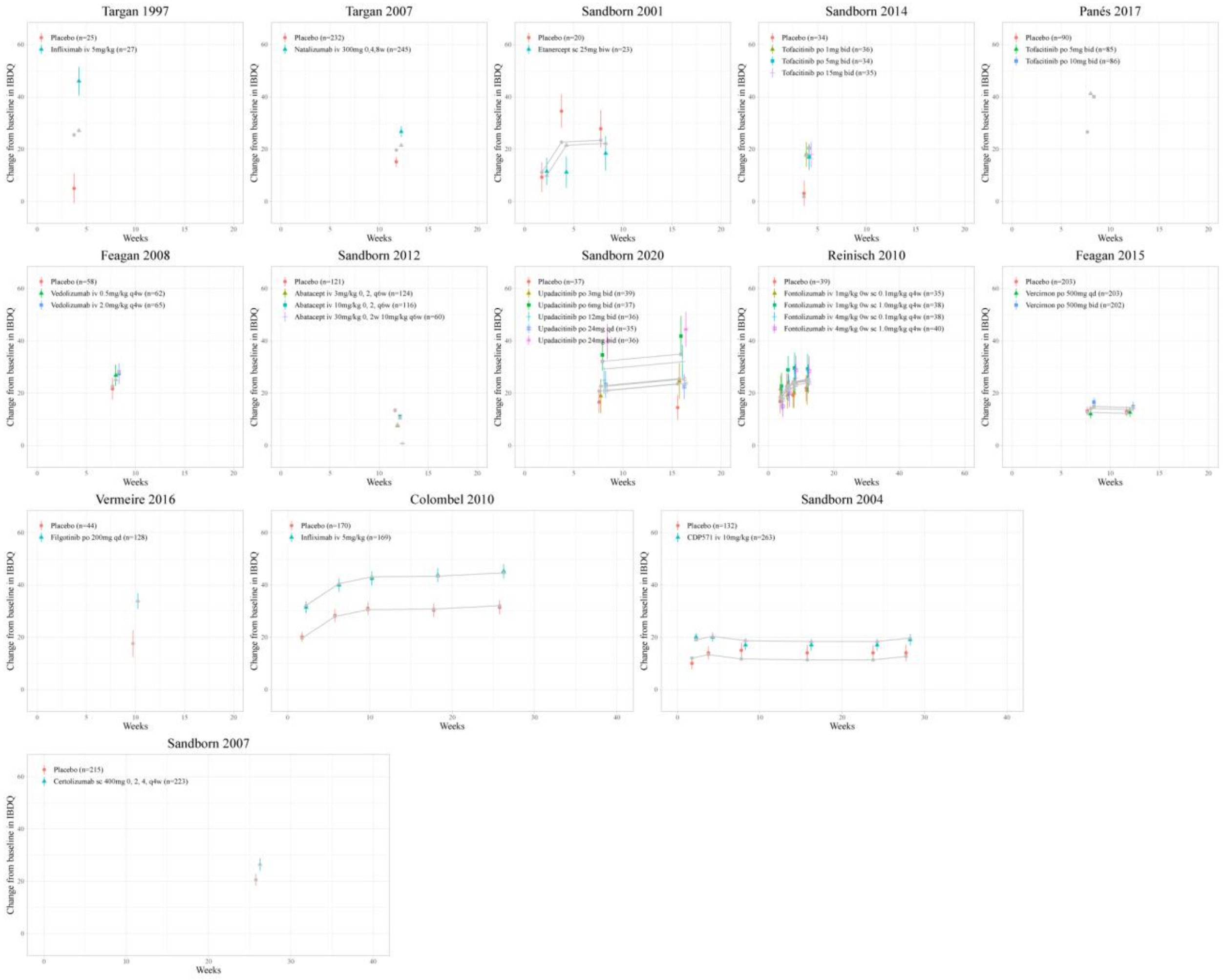

Supplementary Figure 8 *Model fitted time-course plots of IBDQ change from baseline. Color symbols and vertical bars are observed mean and calculated weight of time points; gray symbols and lines are the model predictions. IBDQ, inflammatory bowel disease questionnaire; biw, twice weekly; q4w, once every 4 weeks.*

Diagnostic Plots

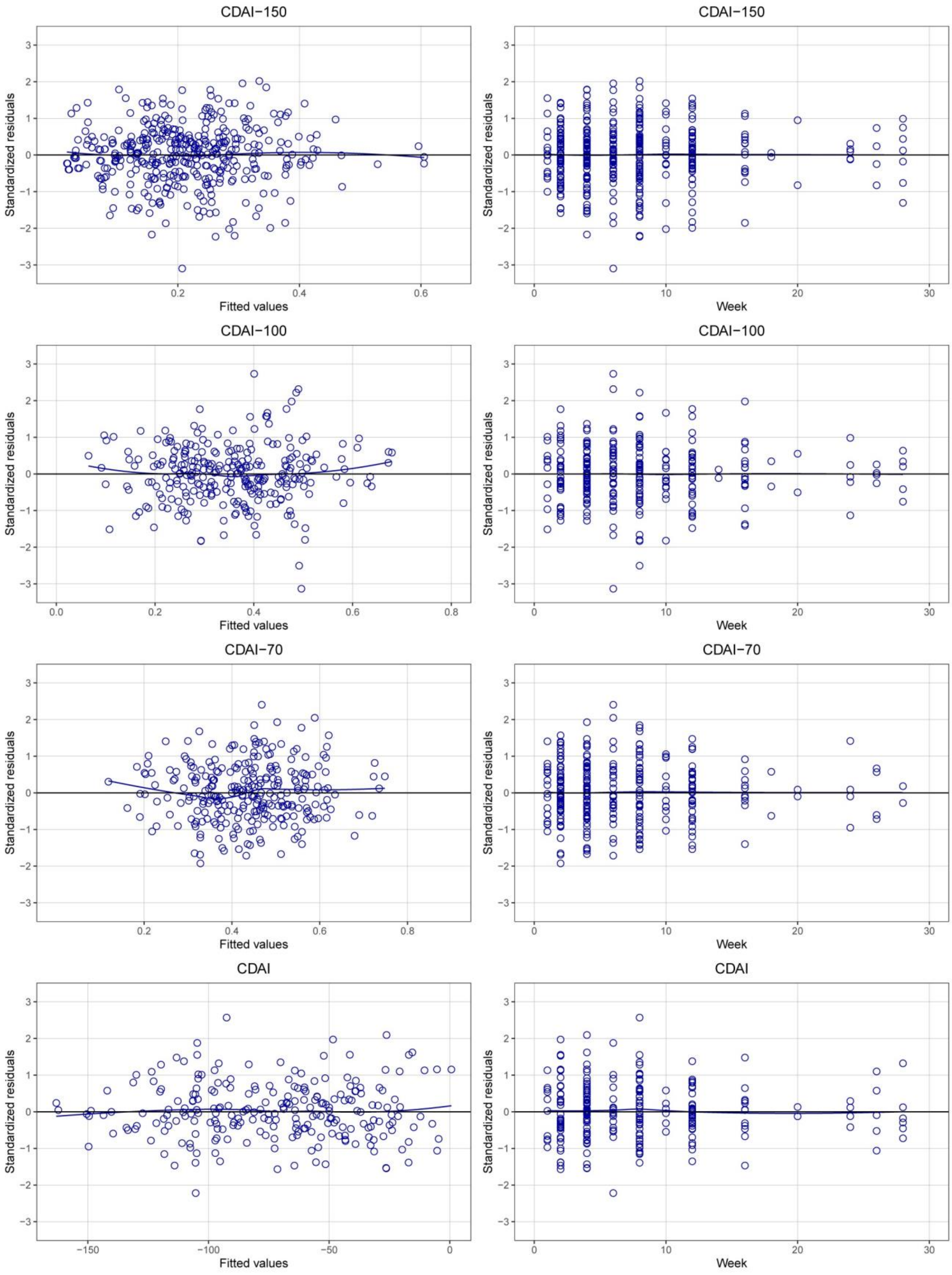

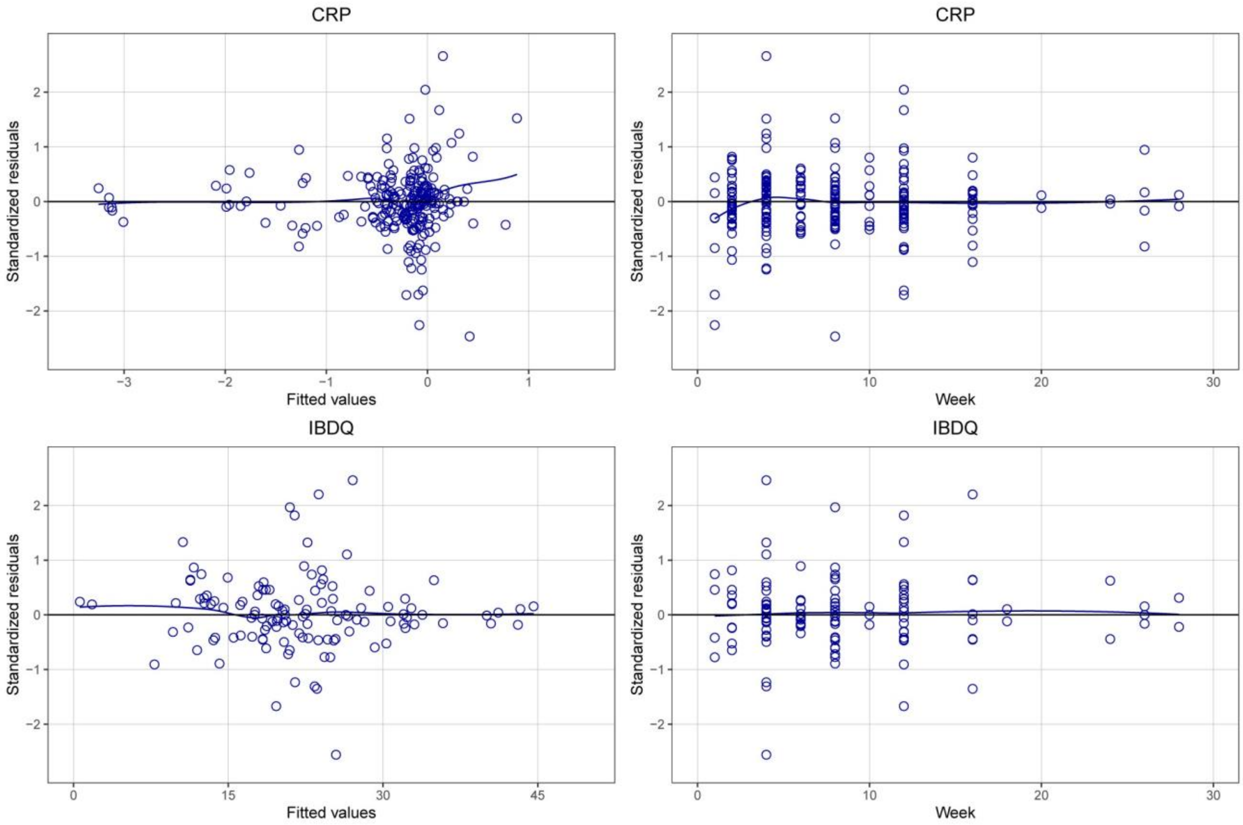

***Supplementary Figure* 9 Diagnostic Plots**

Model Code

Code for CDAI150 model

```
cdai150.14 <- function(group.week,CLASS,FLAG,methodFlag,DOSE,group,week,
                    male,age,dur,CDAI,CRP,eo,eminf,emnat,emcdp,
                    emeta,emcer,emone,emved,emust,emapi,emand,
                    emtof,emfil,emmedi,emupa,emfon,emabr,emver,
                    emlaq,emont,k,kada,kris,kpf1,eCDAI,eCRP)
{
  emrel = 1
  emrel = emrel*(CDAI/mean(CDAI150$CDAI,na.rm = T))**eCDAI
  emrel = emrel*(CRP/mean(CDAI150$CRP,na.rm = T))**eCRP
  etime = 1*(CLASS!=5) + (1-exp(-exp(k)*week))*(CLASS==5)
  emrel = emrel*etime
  efinf = 0 + eminf*(FLAG==2)
  efinf = efinf*emrel
  efnat = 0 + emnat*(FLAG==3)
  efnat = efnat*emrel
  efcdp = 0 + emcdp*(FLAG==4)
  efcdp = efcdp*emrel
  efeta = 0 + emeta*(FLAG==5)
  efeta = efeta*emrel
  efcer = 0 + emcer*(FLAG==6)
  efcer = efcer*emrel
  efada = 0 + (kada*DOSE)*(FLAG==7)
  efada = efada*emrel
  efone = 0 + emone*(FLAG==8)
  efone = efone*emrel
  efved = 0 + emved*(FLAG==9)
```

efved = efved*emrel

efust = 0 + emust*(FLAG==10)

efust = efust*emrel

efapi = 0 + emapi*(FLAG==11)

efapi = efapi*emrel

efand = 0 + emand*(FLAG==12)

efand = efand*emrel

eftof = 0 + emtof*(FLAG==13)

eftof = eftof*emrel

effil = 0 + emfil*(FLAG==14)

effil = effil*emrel

efris = 0 + (kris*DOSE)*(FLAG==16)

efris = efris*emrel

efpf1 = 0 + (kpf1*DOSE)*(FLAG==17)

efpf1 = efpf1*emrel

efmedi = 0 + emmedi*(FLAG==18)

efmedi = efmedi*emrel

efupa = 0 + emupa*(FLAG==19)

efupa = efupa*emrel

effon = 0 + emfon*(FLAG==20)

effon = effon*emrel

efabr = 0 + emabr*(FLAG==21)

efabr = efabr*emrel

efver = 0 + emver*(FLAG==22)

efver = efver*emrel

eflaq = 0 + emlaq*(FLAG==23)

eflaq = eflaq*emrel

efont = 0 + emont*(FLAG==24)

```
  efont = efont*emrel
  emax = 0 + eo + efinf + efnat + efcdp + efeta + efcer + efada + efone + efved + efust + efapi +
efand + eftof + effil + efris + efpf1 + efmedi + efupa + effon + efabr + efver + eflaq + efont
  yp = exp(emax)/(1+exp(emax))
  yp
}
#Note: group.week (each time point in each trial), CLASS (class number of the drug. Placebo is
defined as 0), FLAG (number of each treatment), methodFLAG (number of each route of
administration), group (number of each treatment in each trial)
ncdai150.14 <-
gnls(cdai150~cdai150.14(group.week,CLASS,FLAG,methodFlag,DOSE,group,week,male,age,d
ur,CDAI,
                        CRP,eo,eminf,emnat,emcdp,emeta,emcer,emone,emved,
                        emust,emapi,emand,emtof,emfil,emmedi,
                        emupa,emfon,emabr,emver,emlaq,emont,k,kada,kris,
                        kpf1,eCDAI,eCRP
),
data = CDAI150,
params = list(eo~-1+I(group.week),
              eminf~1,emnat~1,emcdp~1,emeta~1,emcer~1,
              emone~1,emved~1,emust~1,emapi~1,
              emand~1,emtof~1,emfil~1,
              emmedi~1,emupa~1,emfon~1,emabr~1,
              emver~1,emlaq~1,emont~1,k~1,kada~1,kris~1,
              kpf1~1,eCDAI~1,eCRP~1),
start = list(coef(n10),0.6),
correlation = corARMA(form = ~tp1|trialNO/armNO,p=2),
weights=varPower(0.5,form=~fitted(.)*(1-fitted(.))/no,fixed = 0.5),
verbose=T
)
```

Code for CDAI-100 model

```
cdai100.23 <- function(trialNO,armNO,week,FLAG,DOSE,group.week,methodFlag,
                    cdai100,no,tp1,male,age,dur,smok,CDAI,CRP,Regimen,
                    eo,eminf,emnat,emcer,emada,emone,emved,emust,emapi,
                    emtof,emfil,empf1,emmedi,emfon,emver,
                    emlaq,emont,ledada,kupa,eCDAI,eCRP
)
{
  emrel = 1
  emtime = 1
  emrel = emrel*(CDAI/mean(CDAI100$CDAI,na.rm = T))**eCDAI
  emrel = emrel*(CRP/mean(CDAI100$CRP,na.rm = T))**eCRP
  emrel = emrel*emtime
  efinf = 0 + eminf*(FLAG==2)
  efinf = efinf*emrel
  efnat = 0 + emnat*(FLAG==3)
  efnat = efnat*emrel
  efcer = 0 + emcer*(FLAG==4)
  efcer = efcer*emrel
  efada = 0 + ((emada*DOSE)/(DOSE+exp(ledada)))*(FLAG==5)
  efada = efada*emrel
  efone = 0 + emone*(FLAG==6)
  efone = efone*emrel
  efved = 0 + emved*(FLAG==7)
  efved = efved*emrel
  efust = 0 + emust*(FLAG==8)
  efust = efust*emrel
  efapi = 0 + emapi*(FLAG==9)
```

```
  efapi = efapi*emrel
  eftof = 0 + emtof*(FLAG==10)
  eftof = eftof*emrel
  effil = 0 + emfil*(FLAG==11)
  effil = effil*emrel
  efpf1 = 0 + empf1*(FLAG==13)
  efpf1 = efpf1*emrel
  efmedi = 0 + emmedi*(FLAG==14)
  efmedi = efmedi*emrel
  efupa = 0 + (kupa*DOSE)*(FLAG==15)
  efupa = efupa*emrel
  effon = 0 + emfon*(FLAG==16)
  effon = effon*emrel
  efver = 0 + emver*(FLAG==17)
  efver = efver*emrel
  eflaq = 0 + emlaq*(FLAG==18)
  eflaq = eflaq*emrel
  efont = 0 + emont*(FLAG==19)
  efont = efont*emrel
  emax = eo + efinf + efnat + efcer + efada + efone + efved + efust + efapi +
    eftof + effil + efpf1 + efmedi + efupa + effon + efver + eflaq + efont
  yp = exp(emax)/(1+exp(emax))
  yp
}

ncdai100.23 <-
gnls(cdai100~cdai100.23(trialNO,armNO,week,FLAG,DOSE,group.week,methodFlag,
                        cdai100,no,tp1,male,age,dur,smok,CDAI,CRP,Regimen,
```

```
            eo,eminf,emnat,emcer,emada,emone,emved,emust,emapi,
            emtof,emfil,empf1,emmedi,emfon,emver,
            emlaq,emont,ledada,kupa,eCDAI,eCRP
),
data = CDAI100,
params = list(eo~-1+I(group.week),
      eminf~1,emnat~1,emcer~1,emada~1,emone~1,emved~1,emust~1,
      emapi~1,emtof~1,emfil~1,empf1~1,emmedi~1,
      emfon~1,emver~1,emlaq~1,emont~1,ledada~1,kupa~1,eCDAI~1,
      eCRP~1
),
start = c(coef(n17),0.3),
weights=varPower(0.5,form=~fitted(.)*(1-fitted(.))/no,fixed = 0.5),
correlation = corCompSymm(form = ~tp1|group),
verbose = T
)
```

Code for CDAI-70 model

```
cdai70.16<-function(FLAG,group.week,DOSE,Regimen,methodFlag,male,age,dur,CDAI,CRP,
                 eo,eminf,emnat,emcdp,emeta,emcer,emada,emved,emust,
                 emsem,emtof,empf1,emmedi,emupa,emver,emlaq,
                 emont,ledupa,eCDAI,eCRP
)
{
  emrel = 1
  emrel = emrel*(CDAI/mean(CDAI70$CDAI,na.rm = T))**eCDAI
  emrel = emrel*(CRP/mean(CDAI70$CRP,na.rm = T))**eCRP
  efinf = 0 + eminf*(FLAG==2)
  efinf = efinf*emrel
  efnat = 0 + emnat*(FLAG==3)
  efnat = efnat*emrel
  efcdp = 0 + emcdp*(FLAG==4)
  efcdp = efcdp*emrel
  efeta = 0 + emeta*(FLAG==5)
  efeta = efeta*emrel
  efcer = 0 + emcer*(FLAG==6)
  efcer = efcer*emrel
  efada = 0 + emada*(FLAG==7)
  efada = efada*emrel
  efved = 0 + emved*(FLAG==8)
  efved = efved*emrel
  efust = 0 + emust*(FLAG==9)
  efust = efust*emrel
  efsem = 0 + emsem*(FLAG==10)
  efsem = efsem*emrel
```

```
  eftof = 0 + emtof*(FLAG==11)
  eftof = eftof*emrel
  efpf1 = 0 + empf1*(FLAG==13)
  efpf1 = efpf1*emrel
  efmedi = 0 + emmedi*(FLAG==14)
  efmedi = efmedi*emrel
  efupa = 0 + ((emupa*DOSE)/(DOSE+exp(ledupa)))*(FLAG==15)
  efupa = efupa*emrel
  efver = 0 + emver*(FLAG==16)
  efver = efver*emrel
  eflaq = 0 + emlaq*(FLAG==17)
  eflaq = eflaq*emrel
  efont = 0 + emont*(FLAG==18)
  efont = efont*emrel
  # total effect
  emax = 0 + eo + efinf + efnat + efcdp + efeta + efcer + efada + efved +
    efust + efsem + eftof + efpf1 + efmedi + efupa + efver + eflaq +
    efont
  yp =exp(emax)/(1+exp(emax))
  yp
}
ncdai70.16<-gnls(cdai70~
cdai70.16(FLAG,group.week,DOSE,Regimen,methodFlag,male,age,dur,CDAI,CRP,
                        eo,eminf,emnat,emcdp,emeta,emcer,emada,
                        emved,emust,emsem,emtof,empf1,
                        emmedi,emupa,emver,emlaq,emont,ledupa,
                        eCDAI,eCRP
),
```

```
data=CDAI70,
params=list(eo~-1+I(group.week),
        eminf~1,emnat~1,emcdp~1,emeta~1,emcer~1,emada~1,emved~1,
        emust~1,emsem~1,emtof~1,empf1~1,emmedi~1,emupa~1,
        emver~1,emlaq~1,emont~1,ledupa~1,eCDAI~1,eCRP~1
),
start =  c(coef(n13)),
correlation = corAR1(form = ~tp|group),
weights=varPower(0.5,form=~fitted(.)*(1-fitted(.))/no,fixed = 0.5),
verbose=T
)
```

Code for ΔCDAI model

```
cdai27 <- function(trialNO,armNO,week,no,FLAG,DOSE,methodFlag,cdai,SD,group.week,
                 tp1,male,age,dur,CDAI,CRP,
                 eo,emnat,emcdp,emeta,emcer,emada,emved,emtof,
                 emfil,emaba,emris,empf,emmedi,emfon,
                 emabr,emver,k,edada,emCDAI
)
{
  emrel = 1
  emrel = emrel*(CDAI/306.285)**emCDAI
  emtime = 1*(1-exp(-exp(k)*week))
  emrel = emrel*emtime
  efnat = 0 + emnat*(FLAG==3)
  efnat = efnat*emrel
  efcdp = 0 + emcdp*(FLAG==4)
  efcdp = efcdp*emrel
  efeta = 0 + emeta*(FLAG==5)
  efeta = efeta*emrel
  efcer = 0 + emcer*(FLAG==6)
  efcer = efcer*emrel
  efada = 0 + ((emada*(DOSE))/(exp(edada)+DOSE))*(FLAG==7)
  efada = efada*emrel
  efved = 0 + emved*(FLAG==8)
  efved = efved*emrel
  eftof = 0 + emtof*(FLAG==10)
  eftof = eftof*emrel
  effil = 0 + emfil*(FLAG==11)
  effil = effil*emrel
```

```
  efaba = 0 + emaba*(FLAG==13)
  efaba = efaba*emrel
  efris = 0 + emris*(FLAG==14)
  efris = efris*emrel
  efpf = 0 + empf*(FLAG==15)
  efpf = efpf*emrel
  efmedi = 0 + emmedi*(FLAG==16)
  efmedi = efmedi*emrel
  effon = 0 + emfon*(FLAG==17)
  effon = effon*emrel
  efabr = 0 + emabr*(FLAG==18)
  efabr = efabr*emrel
  efver = 0 + emver*(FLAG==19)
  efver = efver*emrel
  emax = eo + efnat + efcdp + efeta + efcer + efada + efved +
    eftof + effil + efaba + efris + efpf + efmedi +
    effon + efabr + efver
  yp = emax
  yp
}
ncdai27 <-
gnls(cdai~cdai27(trialNO,armNO,week,no,FLAG,DOSE,methodFlag,cdai,SD,group.week,
                 tp1,male,age,dur,CDAI,CRP,
                 eo,emnat,emcdp,emeta,emcer,emada,emved,emtof,
                 emfil,emaba,emris,empf,emmedi,emfon,emabr,
                 emver,k,edada,emCDAI
),
data = CDAI,
```

```
params = list(eo~-1+I(group.week),
        emnat~1,emcdp~1,emeta~1,emcer~1,emada~1,emved~1,
        emtof~1,emfil~1,emaba~1,emris~1,empf~1,
        emmedi~1,emfon~1,emabr~1,emver~1,k~1,
        edada~1,emCDAI~1
),
start = c(coef(n8),-2),
correlation = corAR1(form = ~tp1|group),
weights = varFixed(~(SD^2/no)),
verbose = T
)
```

Code for ΔCRP model

```
crp26 <- function(trialNO,armNO,week,FLAG,group.week,methodFlag,crp,SD,
                  DOSE,no,tp1,male,age,dur,CRP,CDAI,eo,eminf,emnat,emcdp,
                  kcer,emada,emved,emust,emsem,emtof,emaba,empf1,kupa,emfon,
                  emver,emlaq,emris,emont,ledpf1,eage,edur,eCRP)
{
  emrel = 1
  emrel = emrel*(age/mean(age,na.rm = T))**eage
  emrel = emrel*(dur/mean(dur,na.rm = T))**edur
  emrel = emrel*(CRP/mean(CRP,na.rm = T))**eCRP
  emtime = 1
  emrel = emrel*emtime
  efinf = 0 + eminf*(FLAG==2)
  efinf = efinf*emrel
  efnat = 0 + emnat*(FLAG==3)
  efnat = efnat*emrel
  efcdp = 0 + emcdp*(FLAG==4)
  efcdp = efcdp*emrel
  efcer = 0 + (kcer*DOSE)*(FLAG==5)
  efcer = efcer*emrel
  efada = 0 + emada*(FLAG==6)
  efada = efada*emrel
  efved = 0 + emved*(FLAG==7)
```

```
efved = efved*emrel

efust = 0 + emust*(FLAG==8)

efust = efust*emrel

efsem = 0 + emsem*(FLAG==9)

efsem = efsem*emrel

eftof = 0 + emtof*(FLAG==10)

eftof = eftof*emrel

efaba = 0 + emaba*(FLAG==11)

efaba = efaba*emrel

efpf1 = 0 + ((empf1*DOSE)/(DOSE+exp(ledpf1)))*(FLAG==12)

efpf1 = efpf1*emrel

efupa = 0 + (kupa*DOSE)*(FLAG==13)

efupa = efupa*emrel

effon = 0 + emfon*(FLAG==14)

effon = effon*emrel

efver = 0 + emver*(FLAG==15)

efver = efver*emrel

eflaq = 0 + emlaq*(FLAG==16)

eflaq = eflaq*emrel

efris = 0 + emris*(FLAG==17)

efris = efris*emrel

efont = 0 + emont*(FLAG==18)

efont = efont*emrel
```

```
  yp = eo + efinf + efnat + efcdp + efcer + efada + efved + efust + efsem +
    eftof + efaba + efpf1 + efupa + effon + efver + eflaq + efris + efont
  yp
}

ncrp26 <- gnls(crp~crp26(trialNO,armNO,week,FLAG,group.week,methodFlag,crp,SD,
                   DOSE,no,tp1,male,age,dur,CRP,CDAI,eo,eminf,emnat,emcdp,
                   kcer,emada,emved,emust,emsem,emtof,emaba,empf1,kupa,emfon,
                   emver,emlaq,emris,emont,ledpf1,eage,edur,eCRP),
         data = CRP,
         params = list(eo~-1+I(group.week),
                     eminf~1,emnat~1,emcdp~1,kcer~1,emada~1,
                     emved~1,emust~1,emsem~1,emtof~1,emaba~1,
                     empf1~1,kupa~1,emfon~1,emver~1,emlaq~1,
                     emris~1,emont~1,ledpf1~1,eage~1,edur~1,
                     eCRP~1),
         start = c(coef(n22),-1),
         correlation = corARMA(form = ~tp1|trialNO/armNO,p=2),
         weights = varFixed(~(SD^2)/no),
         verbose = T
)
```

Code for ΔIBDQ model

```
ibdq23 <- function(trialNO,armNO,DOSE,methodFlag,FLAG,week,no,tp1,group.week,
                 ibdq,SD,male,age,dur,CDAI,CRP,IBDQ,eo,eminf,emnat,emcdp,
                 emeta,emcer,kada,emved,emsem,emapi,emtof,emfil,
                 emaba,kris,emupa,emfon,emver,edur
)
{
  emrel = 1
  emrel = emrel*(dur/mean(dur,na.rm = T))**edur
  emtime = 1
  emrel = emrel*emtime
  efinf = 0 + eminf*(FLAG==2)
  efinf = efinf*emrel
  efnat = 0 + emnat*(FLAG==3)
  efnat = efnat*emrel
  efcdp = 0 + emcdp*(FLAG==4)
  efcdp = efcdp*emrel
  efeta = 0 + emeta*(FLAG==5)
  efeta = efeta*emrel
  efcer = 0 + emcer*(FLAG==6)
  efcer = efcer*emrel
  efada = 0 + (kada*DOSE)*(FLAG==7)
  efada = efada*emrel
```

```
efved = 0 + emved*(FLAG==8)

efved = efved*emrel

efsem = 0 + emsem*(FLAG==10)

efsem = efsem*emrel

efapi = 0 + emapi*(FLAG==11)

efapi = efapi*emrel

eftof = 0 + emtof*(FLAG==12)

eftof = eftof*emrel

effil = 0 + emfil*(FLAG==13)

effil = effil*emrel

efaba = 0 + emaba*(FLAG==14)

efaba = efaba*emrel

efris = 0 + (kris*DOSE)*(FLAG==15)

efris = efris*emrel

efupa = 0 + emupa*(FLAG==16)

efupa = efupa*emrel

effon = 0 + emfon*(FLAG==17)

effon = effon*emrel

efver = 0 + emver*(FLAG==18)

efver = efver*emrel

emax = eo + efinf + efnat + efcdp + efeta + efcer + efada+ efved +
  efsem + efapi + eftof + effil + efaba + efris + efupa +
  effon + efver
```

```
  emax
}

nibdq23 <- gnls(ibdq~ibdq23(trialNO,armNO,DOSE,methodFlag,FLAG,week,no,tp1,
group.week,
                    ibdq,SD,male,age,dur,CDAI,CRP,IBDQ,eo,eminf,emnat,emcdp,
                    emeta,emcer,kada,emved,emsem,emapi,emtof,emfil,
                    emaba,kris,emupa,emfon,emver,edur),
          data = IBDQ,
          params = list(eo~-1+I(group.week),eminf~1,emnat~1,emcdp~1,
                      emeta~1,emcer~1,kada~1,emved~1,
                      emsem~1,emapi~1,emtof~1,emfil~1,emaba~1,
                      kris~1,emupa~1,emfon~1,emver~1,edur~1),
          start = c(coef(n22)),
          correlation = corARMA(form = ~tp1|group,p=1),
          weights = varFixed(~(SD^2)/no),
          verbose = T

)
```
